# Spatial transcriptomics of the aging mouse brain reveals origins of inflammation in the white matter

**DOI:** 10.1038/s41467-025-58466-2

**Published:** 2025-04-04

**Authors:** Lin Wang, Chang-Yi Cui, Christopher T. Lee, Monica Bodogai, Na Yang, Changyou Shi, Mustafa O. Irfanoglu, James R. Occean, Sadia Afrin, Nishat Sarker, Ross A. McDevitt, Elin Lehrmann, Shahroze Abbas, Nirad Banskota, Jinshui Fan, Supriyo De, Peter Rapp, Arya Biragyn, Dan Benjamini, Manolis Maragkakis, Payel Sen

**Affiliations:** 1 Laboratory of Genetics and Genomics, National Institute on Aging, NIH, Baltimore, MD USA; 2 Laboratory of Molecular Biology and Immunology, National Institute on Aging, NIH, Baltimore, MD USA; 3 Quantitative Medical Imaging Section, National Institute of Biomedical Imaging and Bioengineering, NIH, Bethesda, MD USA; 4 Comparative Medicine Section, National Institute on Aging, NIH, Baltimore, MD USA; 5 Computational Biology and Genomics Core, Laboratory of Genetics and Genomics, National Institute on Aging, NIH, Baltimore, MD USA; 6 Center for Alzheimer’s and Related Dementia, National Institute on Aging, NIH, Bethesda, MD USA; 7 Laboratory of Behavioral Neuroscience, National Institute on Aging, NIH, Baltimore, MD USA; 8 Present Address: Department of Biochemistry and Molecular Biology, University of Maryland School of Medicine, Baltimore, USA; 9 Present Address: Cancer Biology Program, Stanford University School of Medicine, Stanford, CA USA

**Keywords:** Epigenetics in the nervous system, Cognitive ageing, Genetics of the nervous system

## Abstract

To systematically understand age-induced molecular changes, we performed spatial transcriptomics of young, middle-aged, and old mouse brains and identified seven transcriptionally distinct regions. All regions exhibited age-associated upregulation of inflammatory mRNAs and downregulation of mRNAs related to synaptic function. Notably, aging white matter fiber tracts showed the most prominent changes with pronounced effects in females. The inflammatory signatures indicated major ongoing events: microglia activation, astrogliosis, complement activation, and myeloid cell infiltration. Immunofluorescence and quantitative MRI analyses confirmed physical interaction of activated microglia with fiber tracts and concomitant reduction of myelin in old mice. In silico analyses identified potential transcription factors influencing these changes. Our study provides a resourceful dataset of spatially resolved transcriptomic features in the naturally aging murine brain encompassing three age groups and both sexes. The results link previous disjointed findings and provide a comprehensive overview of brain aging identifying fiber tracts as a focal point of inflammation.

## Abstract (web summary)

This study profiles the spatial transcriptomic landscape of mouse brain coronal sections across three age groups and both sexes. Here, authors show a strong female-biased pro-inflammatory gene signature in white matter fiber tracts.

## Introduction

It is estimated that 50 million people worldwide have neurodegenerative diseases, and by 2050, this number will triple^1^. Age is the strongest risk factor for many neurodegenerative conditions, including late-onset sporadic Alzheimer’s disease and related dementias (ADRD), Parkinson’s disease (PD), amyotrophic lateral sclerosis (ALS), etc^2^. Brain aging is accompanied by gross morphological changes and cognitive deficits that likely contribute to disease onset^3^. Mapping the progressive cellular and molecular alterations that drive age-related cognitive decline is thus critical for designing ameliorative therapies. Importantly, there is an emphasis on more diagnostic (after disease) over pre-diagnostic (aging but no disease) experimentations that prevent our understanding of disease incidence mechanisms.

Aging and age-related diseases show spatial bias in most complex tissues, including the brain, due to differences in blood flow, metabolic demands, and chemoattractant or chemorepellent signals. Recent studies have only begun to reveal some interesting spatial signatures of aging. Cells responding to acute or chronic injury have been shown to organize into cellular neighborhoods and reside in niches that depend on the presence or absence of other cells. For example, in the aging liver, the zone 3 pericentral hepatocytes undergo accelerated aging^4^ and have the greatest neoplastic potential^5^. In the injured kidney, immune-active altered cellular niches form in the proximal tubules and thick ascending limbs^6^. In aged skeletal muscle, cell types are mislocalized and show signs of both early injury and late regeneration^7^. In AD brains, transcriptional changes occur around amyloid plaques^8^.

A common spatial signature across tissues during injury or aging is the activation of a localized immune response (sterile inflammation) concurrent with productive or maladaptive repair. The brain is an otherwise immune-privileged organ due to the presence of the blood–brain barrier (BBB), a tightly controlled system of blood vessels that allow only select agents to pass through. Thus, immune activation in the aging or diseased brain is of particular interest and may contribute to the rapid loss of neurons and cognitive impairment. Several brain-resident cell types have been implicated in this pro-inflammatory response, including microglia, astrocytes, and oligodendrocytes^9^. Alternatively, or simultaneously, age-related BBB deterioration can cause peripheral immune cell infiltration and an aggravated inflammatory response^10^.

To comprehensively understand brain aging at the cellular and molecular level, we undertook a deep spatiotemporal profiling of aging coronal brain sections from both male and female mice. Our data captured previously known and unknown transcriptomic changes as a function of age and sex and identified the white matter fiber tract (hereon fiber tract) as a key anatomical region vulnerable to age-related inflammation. We present our work as a resource to the community for interrogation of age-related disease mechanisms in the brain.

## Results

### Profiling the aging murine brain using spatial transcriptomics

**Fig. 1:**
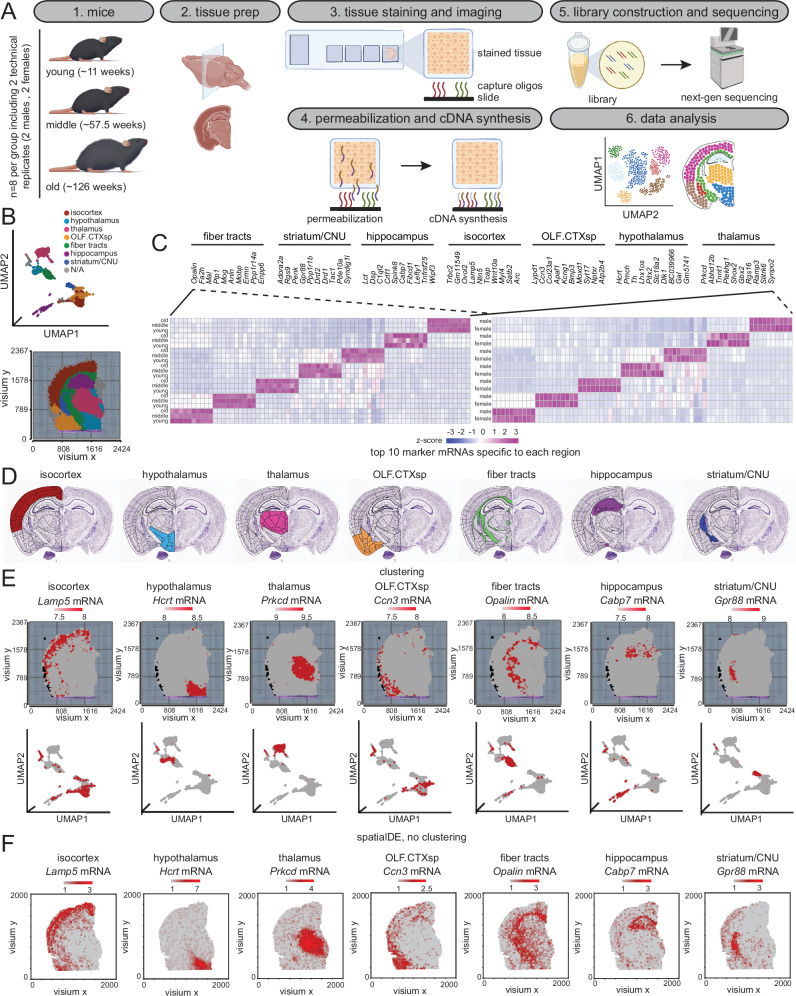
Spatial transcriptomic profiles partition the brain into discrete anatomical regions. **A** Overview of ST workflow using the 10X Genomics Visium platform. **B** Representative Visium image of O2FB (top, *n* = 3419 spots) showing spatial transcriptional profiles colored by cluster-based annotation (bottom). **C** Heatmap of top ten marker mRNAs (count mean values) in young, middle-aged, and old (left) or male and female (right) groups unique to each region. **D** Allen Brain Atlas P56^14^ coronal section images from http://mouse.brain-map.org/static/atlas showing the spatial locations of the seven major regions identified in (**C**). The relevant links are: Isocortex: https://atlas.brain-map.org/atlas?atlas=1&plate=100960084#atlas=1&plate=100960084&resolution=20.94&x=5519.9993896484375&y=3904.8334765434265&zoom=-4&structure=315&z=6. Hypothalamus: https://atlas.brain-map.org/atlas?atlas=1&plate=100960084#atlas=1&plate=100960084&resolution=20.94&x=5519.9993896484375&y=3904.8334765434265&zoom=-4&structure=1097&z=6. Thalamus: https://atlas.brain-map.org/atlas?atlas=1&plate=100960084#atlas=1&plate=100960084&resolution=20.94&x=5519.9993896484375&y=3904.8334765434265&zoom=-4&structure=549&z=6. OLF.CTXsp: https://atlas.brain-map.org/atlas?atlas=1&plate=100960084#atlas=1&plate=100960084&resolution=20.94&x=5519.9993896484375&y=3904.8334765434265&zoom=-4&structure=698&z=6. https://atlas.brain-map.org/atlas?atlas=1&plate=100960084#atlas=1&plate=100960084&resolution=20.94&x=5519.9993896484375&y=3904.8334765434265&zoom=-4&structure=703&z=6. Fiber tract: https://atlas.brain-map.org/atlas?atlas=1&plate=100960084#atlas=1&plate=100960084&resolution=20.94&x=5519.9993896484375&y=3927.9999804496765&zoom=-4&structure=1009&z=6. Hippocampus: https://atlas.brain-map.org/atlas?atlas=1&plate=100960084#atlas=1&plate=100960084&resolution=20.94&x=5519.9993896484375&y=3904.8334765434265&zoom=-4&structure=1080&z=6. CNU/striatum: https://atlas.brain-map.org/atlas?atlas=1&plate=100960084#atlas=1&plate=100960084&resolution=20.94&x=5519.9993896484375&y=3904.8334765434265&zoom=-4&structure=623&z=6. **E** Visium images (top) and UMAP (bottom) visualization of seven major regions showing the expression of representative well-known region-specific marker mRNAs in an old female sample (O2FB). Numbers in the color scale reflect the number of UMIs detected for the specified mRNA for each spot. **F** Same as (**E**) except profiles generated using a non-cluster-based method, SpatialDE (see Methods). Illustration credit for parts of (**A**) goes to Endosymbiont GmbH, and BioRender. Sen, P. (2025) https://BioRender.com/u24a349. Source data are provided as a Source Data file.

Spatial transcriptomics (ST) can reveal mRNA differences, paracrine signaling, and cellular networks not captured in averaged spatially naive analyses of homogenized biopsies^11^. This prospect of multi-dimensional in situ output motivated us to use ST in aging mouse brains across 3 age groups (young (Y, ~11 weeks), middle-aged (M, ~57.5 weeks), and old (O, ~126 weeks)) and both sexes of C57BL/6JN mice (Supp. Data 1). Each age group contained four biological replicates (males (M) *n* = 2 and females (F) *n* = 2) and 2 technical replicates (A or B) i.e., independent, succeeding coronal sections from the same OCT block, for a total of 24 samples (Fig. 1A, steps 1 and 2).

We used the Visium ST platform (10X Genomics) to measure total mRNA in intact tissue sections. The Visium slides contain spatial barcodes that retrospectively link gene activity to its location. Our choice for the Visium platform was motivated by greater tissue coverage, resolution, and throughput compared to existing techniques such as laser microdissection RNA-seq or in situ hybridization^12^. Prior to working with real samples, we performed a careful permeabilization optimization on one young sample (Supp. Fig. 1A). Mouse brain sections (16 µm) were fixed, stained, and permeabilized for different lengths of time (3–30 min) on a tissue optimization slide (see Methods). mRNA released during permeabilization bound to capture probes on the optimization slide with minimum lateral diffusion. cDNA was generated using fluorescently labeled nucleotides to enable visualization by microscopy. After fluorescence imaging, we selected 30 min as the optimum permeabilization time as it showed the maximum fluorescence signal and the lowest signal diffusion. The brightfield image confirmed that the lack of or suboptimal fluorescence signal at other time points was due to insufficient permeabilization and not missing tissue. This optimized permeabilization time was applied to all experimental sections to release mRNA on a Visium spatial gene expression slide.

Each Visium spatial gene expression slide contains 4 capture areas, with each area containing ~5000 barcoded spots, being about 55 µm in diameter, and covering ~5–25 cells per spot for a 16 µm section. We randomized tissue sections and hemispheres from each age group over several gene expression slides (Supp. Data 2). Tissue sections were first stained with Hematoxylin-Eosin (H&E), followed by cDNA synthesis, amplification, barcoding, and library construction (see Methods). Following this demonstrated protocol, we obtained high-quality spatially resolved RNA-seq data from these 24 samples and sequenced them on the NovaSeq™ 6000 platform achieving an average coverage of 171 million read pairs per sample, in line with recommendations (Fig. 1A, steps 3–6). Representative bioanalyzer traces post-cDNA amplification and library PCR are shown in Supp Fig. 1B. We observed the expected average fragment size and yield of cDNA (top) and libraries (bottom) for each sample. After next generation sequencing, we processed the data through Space Ranger (10X Genomics) which aligned, extracted barcodes, counted unique molecular identifiers (UMIs), and compiled a feature-barcode matrix for each sample. Space Ranger was further used to assess quality control metrics which revealed an average of 3590 spots under the tissue (~72% coverage of 5000 spots), including an average of 45,995 mean reads per spot and 4179 median genes per barcode (Supp. Data 2).

### Spatial transcriptomic profiles partition the brain into discrete anatomical regions

We next imported the feature-barcode matrix from Space Ranger into Partek Flow Genomic Analysis Software (Partek Inc.)^13^ for further analysis. The data were first preprocessed by filtering out low-quality spots, removing lowly expressed mRNAs (value ≤1.0 in at least 99.0% of spots), defining thresholds for mitochondrial (0–35%) and ribosomal (0–14%) RNA counts (Supp. Fig. 1C, D), variance stabilizing by log normalization and finally normalizing RNA counts to sequencing depth (counts per million or CPM) (Supp. Fig. 1E). This filtered list contained 10109 mRNAs (Supp. Data 3). A correlation analysis of counts across these ~10,000 mRNAs showed high concordance (Pearson’s *r* = 0.93–1) between technical and biological replicates and even among different age groups suggesting few global transcriptional changes in the brain with age.

We next performed principal component analysis (PCA) to reduce the dimensionality of the data followed by graph-based clustering using the Louvain algorithm (Fig. 1B top). We annotated the clusters to the captured H&E image of each sample and fine-tuned the fit by adjusting the resolution and manually correcting any misidentified spots (Fig. 1B bottom). Remarkably, the clusters neatly classified into seven different brain anatomical regions as seen in the Allen Mouse Brain Atlas^14^; striatum/cerebral nuclei (CNU), isocortex, olfactory region with cortical subplate (OLF.CTXsp), fiber tracts, hippocampus, hypothalamus, and thalamus (Fig. 1C, D). The top ten marker mRNAs unique to each cluster are shown for young, middle-aged, and old age (Fig. 1C, left) and male and female (Fig. 1C, right) groups. Some marker mRNAs based on the clustering into seven anatomical regions are annotated to the histological sections and shown in Fig. 1E. We used an orthogonal and non-clustering-based approach called SpatialDE^15^, to map mRNA profiles across the mouse brain. SpatialDE uses a Bayesian hierarchical model and assumes that the mRNA levels vary smoothly across the tissue, and that nearby cells are more similar in their expression profiles than distant cells. SpatialDE also identified distinct transcriptomic profiles across the seven anatomical regions of the mouse brain (Fig. 1F).

Overall, both approaches confirmed that spatial transcriptomic profiles partition the brain into distinct anatomical regions which then allowed us to compare mRNA profiles in these different regions across age and sex groups. The number of spots covered by each region was proportional to its size (Supp. Fig. 1F), and there were no observable differences between the groups based on age (young, middle, and old) (Supp. Fig. 1G) or sex (male and female) (Supp. Fig. 1H).

### Anatomical regions of the brain display both shared and unique sets of age-correlated mRNAs

**Fig. 2:**
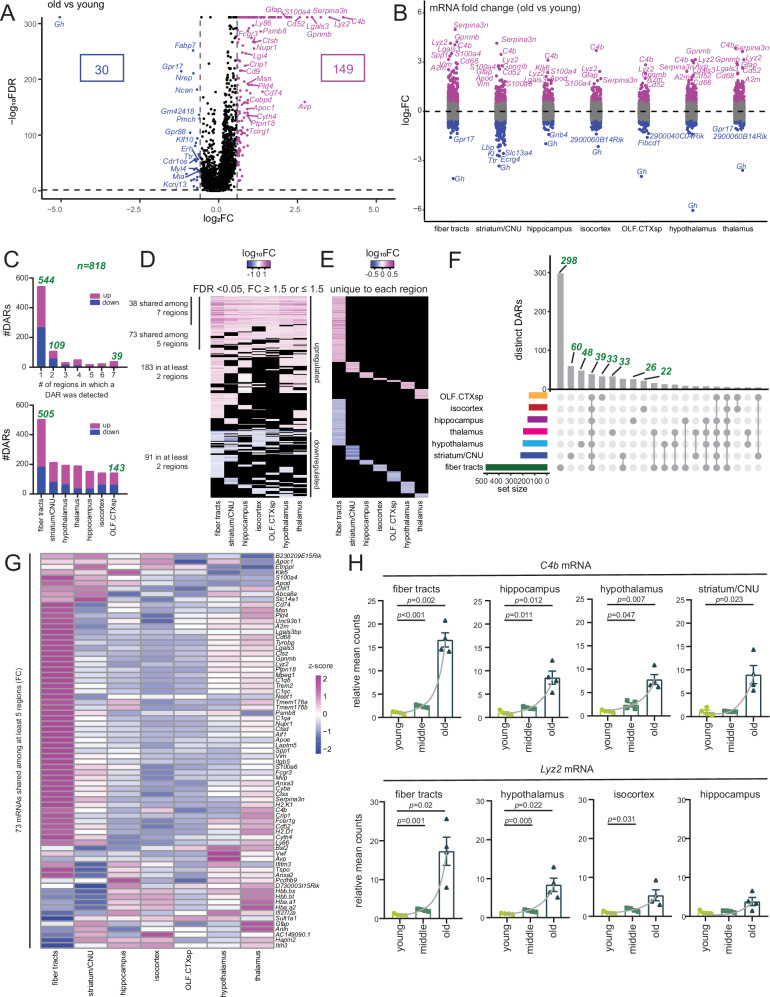
Anatomical regions of the brain display both shared and unique sets of age-correlated mRNAs. **A** Volcano plot showing -log_10_(FDR) and log_2_(FC) values for all 10109 mRNAs with 149 significantly upregulated (purple dots; FDR <0.05, FC ≥1.5) and 30 significantly downregulated (blue dots; FDR <0.05, FC ≤−1.5) DARs in old vs young. **B** Strip chart showing log_2_FC value for all 10109 mRNAs across seven regions with significantly upregulated and significantly downregulated (as in (**A**)) DARs highlighted in each region. **C** Bar plot showing the number of regions in which a given DAR was detected (top), and number of DARs that were either significantly upregulated or downregulated (as in (**A**)) with age in each region (bottom). **D** Heatmap showing log_10_FC values for age-correlated mRNAs across at least two brain regions. **E** Heatmap showing log_10_FC values for age-correlated mRNAs unique to each region. **F** Upset plot of age-correlated mRNAs shared across and unique to each region (each matrix column represents either DARs specific to a region (single circle with no vertical lines) or DARs shared between regions (with a vertical line connecting regions that share a given DAR). Vertical bar plots on top display the number of DARs in each combination of regions. Horizontal bar plots on the left display the total number of DARs for a given region. Sets with ≥5 mRNAs are shown. **G** Heatmap showing FC (old vs young) values for 73 mRNAs upregulated with age and shared between at least five regions. **H** Bar plot of *C4b* (top) and *Lyz2* (bottom) expression at indicated regions. Each dot shows the average expression of the two technical replicates for each mouse relative to the mean of the young group, equivalent to *n* = 4 biological replicates per group. Data were summarized as mean ± standard error of the relative mean counts. *p* values are reported from an unpaired two-tailed *t*-test with Welch’s correction comparing old vs. young and middle vs. young. Note that the slope of the gray lines connecting the means is the highest in fiber tracts. Source data are provided as a Source Data file.

At first pass, we used ANOVA to identify differentially abundant mRNAs across all brain regions (akin to pseudobulk transcriptomics) in the three age groups. We classified an mRNA to be differentially abundant (DAR) in one age group if the false discovery rate from the Benjamini–Hochberg method (FDR) <0.05 and fold change (FC) ≥1.5 (upregulated) or ≤–1.5 (downregulated) (Fig. 2A, B, Supp. Fig. 2A, B, and Supp. Data 4). DARs upregulated with age showed a distinct immune activation signature and included mRNAs related to antigen processing and presentation such as proteasome 20S subunit beta 8 (*Psmb8* mRNA) and cathepsin H (*Ctsh* mRNA), microglia activation such as allograft inflammatory factor (*Aif1* mRNA), galectin-3 (*Lgals3* mRNA), cluster of differentiation 68 (*Cd68* mRNA), triggering receptor expressed on myeloid cells 2 (*Trem2* mRNA), tyrosine kinase-binding protein (*Tyrobp* mRNA), lysozyme 2 (*Lyz2* mRNA), glycoprotein non-metastatic melanoma protein B-like protein (*Gpnmb* mRNA), and secreted phosphoprotein 1 (*Spp1* mRNA), complement system activation such as complement proteins 1q (*C1q* mRNA) and 4b (*C4b* mRNA), reactive astrogliosis such as serine peptidase inhibitor, clade A member 3N (*Serpina3n* mRNA), S100 calcium binding protein A4 (*S100a4* mRNA), glial fibrillary acidic protein (*Gfap* mRNA) and vimentin (*Vim* mRNA), as well as neuronal excitation and synaptic plasticity such as leucine-rich glioma inactivated 4 (*Lgi4* mRNA), cluster of differentiation 9 (*Cd9* mRNA), S100 calcium binding protein A4 (*S100a4* mRNA) and nuclear protein transcriptional regulator 1 (*Nupr1* mRNA). By contrast, DARs downregulated with age were related to brain development and adult neurogenesis, such as growth hormone (*Gh* mRNA), fatty acid binding protein 7 (*Fabp7* mRNA), neuronal regeneration-related protein (*Nrep* mRNA), and Kruppel-like factor 10 (*Klf10* mRNA). The expanded Gene Ontology (GO) terms associated with these age-related DARs are shown in Supp. Fig. 2C–E for three-way comparison between young, middle-aged, and old samples. When plotting the pseudobulk DARs across the three age groups, it was evident that a majority were gradually upregulated with age (Supp. Fig. 3, clusters 1, 2, and 4, *n* = 149), with much of the increase occurring in the oldest age group. A smaller group of mRNAs were gradually downregulated with age (Supp. Fig. 3, clusters 9–10, *n* = 23).

We next queried whether age-related DARs showed any spatial bias in the mouse brain. Even when visualizing all 10109 mRNAs at once, it was evident that different anatomical regions of the brain were unique in their mRNA signatures (Supp. Fig. 2F–G). The strip chart in Fig. 2B displays DARs in each of the seven brain regions. A total of 818 DARs, significantly affected by aging, were identified in at least one region. About 544 of these DARs were uniquely assigned to only one region, while only 39 were shared across all brain regions suggesting strong spatial preference in the aging brain transcriptome (Fig. 2C, top). Interestingly, 505 DARs were traced to the fiber tract region (Fig. 2C, bottom), a region that is composed largely of myelinated axons and responsible for facilitating communication across brain regions. Some prominent tracts visible in the coronal section are the corticospinal tract and the corpus callosum. The corticospinal tract is the largest at the rostrocaudal level, encompassing the internal capsule and cerebral peduncle^16^. This tract connects primary motor and somatosensory cortices with subcortical structures, en route to the spinal cord. The corpus callosum connects interhemispheric cortical regions, and shows age-related decline in size, microstructural integrity, and associated cognitive function^17^. Most upregulated DARs were shared across brain regions (Fig. 2D) and were related to immune activation; 38 upregulated DARs were shared across all seven regions, 73 across at least five regions, and 183 in at least two regions, suggesting a coordinated change (Supp. Data 5). Approximately 91 downregulated DARs shared across at least two brain regions were related to neuropeptide signaling and nervous system development (Supp. Data 5). Among the uniquely upregulated DARs, the fiber tracts showed the highest number (*n* = 177, Fig. 2E, Supp. Data 5), with many DARs being related to the extracellular matrix, wound healing, cell motility, and migration suggestive of ongoing injury and repair. Similarly, fiber tracts also showed the most downregulated DARs (*n* = 126, Fig. 2E and Supp. Data 5). The upset plot in Fig. 2F shows a summarized matrix layout of DARs shared across and specific to each region.

Overall, our data show that different brain regions have strong spatial preference in age-related transcriptomic changes, although there are some shared features. Of interest, the fiber tract region appears to show the strongest changes in mRNA with age.

### Aging fiber tracts exhibit signs of immune activation

In the DARs that showed shared regulation across brain regions (Supp. Data 4), it was intriguing to note that they undergo the highest FC (old vs young) in the fiber tract (Fig. 2G). Figure 2H demonstrates substantial region-dependence of two strong DARs, *C4b* and *Lyz2*, with strong age-related upregulation at fiber tracts compared to other regions. By contrast, the unique DARs showed a much weaker FC (note the log_10_ scale in Fig. 2D, E). The annotated Visium images of some shared upregulated DARs are shown in Supp. Fig. 4A–R highlighting the spatial bias at fiber tracts.

We next performed a region-by-region gene set enrichment analysis (GSEA) to query the functional relevance of mRNAs that are strongly enriched with age (Supp. Fig. 5 and Supp. Data 6). All seven regions showed an enrichment in immune-related pathways in the old. By contrast, pathways related to synaptic function, particularly in the thalamus and hypothalamus and evident at the pseudobulk level (Supp. Fig. 2C–E) were strongly depleted. Given that fiber tracts show the strongest FC in shared age-associated mRNAs (Fig. 2G), we conclude that immune-modulatory mRNAs are markedly increased in the fiber tracts of aging brains.

**Fig. 3:**
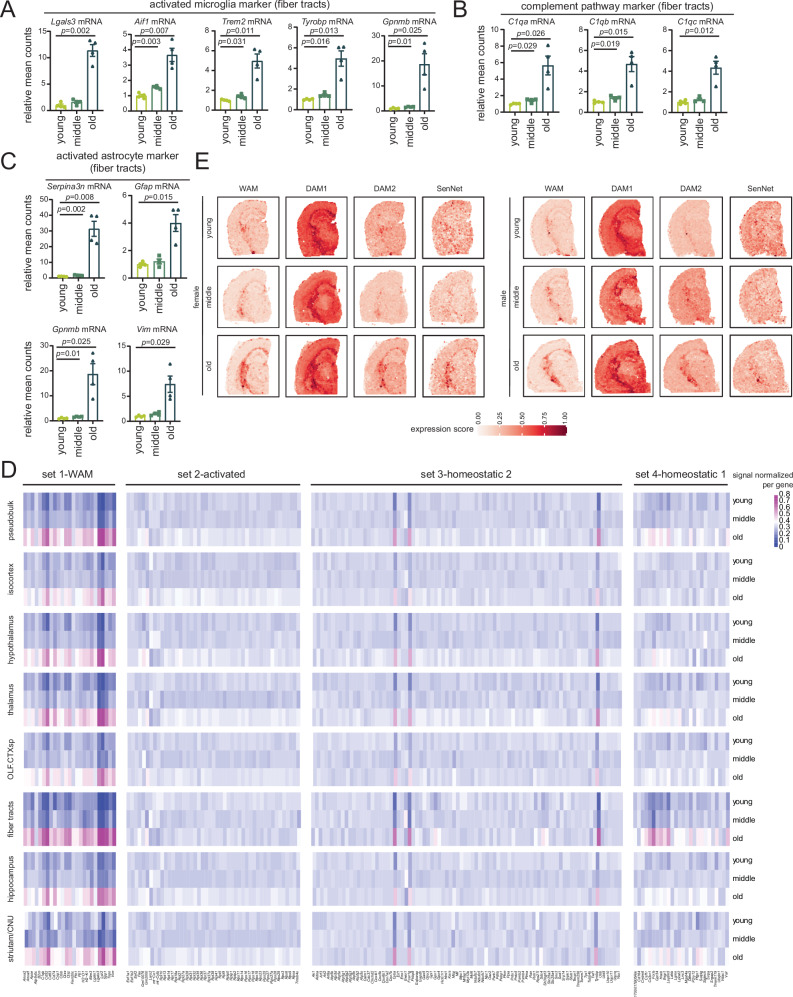
Aging fiber tracts are enriched in WAM and DAM2 signatures. **A** Bar plot of activated microglia marker mRNAs *Lgals3*, *Aif1*, *Trem2*, *Tyrobp*, and *Gpnmb*, **B** complement pathway marker mRNAs *C1qa*, *C1qb*, and *C1qc*, and **C** activated astrocyte marker mRNAs *Serpina3n*, *Gfap*, *Gpnmb*, and *Vim* in fiber tracts of young, middle, and old mice. Each dot shows the average expression of the two technical replicates for each mouse relative to the mean of the young group, equivalent to *n* = 4 biological replicates per group. Data were summarized as mean ± standard error of the relative mean counts. *p* values are reported from an unpaired two-tailed *t*-test with Welch’s correction comparing middle vs. young and old vs. young. **D** Heatmap showing average expression in young, middle, and old groups of four mRNA sets (set 1–4) from ref. ^18^. Values in the color scale are signals normalized on a per mRNA basis. **E** Visualization of average expression of WAM (*n* = 29), DAM1 (*n* = 77), top DAM2 (*n* = 92), and SenNet (*n* = 17) mRNA sets in young (Y1MB, Y2FB), middle (M2MA, M1FB), and old (O2MB, O1FB) mice using SPATA (see Methods). The WAM mRNA set is from ref. ^18^, the DAM1 and DAM2 mRNA sets are from ref. ^19^, and the SenNet mRNA set is from ref. ^20^ (Supp. Data 7). Values in the color scale are the expression scores of the mRNA sets. Source data are provided as a Source Data file.

Among the immune-modulatory mRNAs upregulated throughout the brain and particularly the fiber tract, several mRNAs related to microglia activation (*Lgals3*, *Aif1*, *Trem2*, *Tyrobp*, *Lyz2*, *Gpnmb* mRNAs), complement system activation (*C1q*, *C4b* mRNAs), and reactive astrogliosis (*Serpina3n*, *Gfap*, *Vim*, *Gpnmb* mRNAs) were evident (Figs. 2H, 3A–C). Among those previously reported, LGALS3 is strongly expressed in white matter-associated microglia (WAM)^18^ and forms nodular clusters in the corpus callosum of aged mice brains, while TREM2 classifies both WAM^18^ and the rare disease-associated microglia subtype stage 2 (DAM2)^19^, spatially associated with sites of AD pathology (Supp. Data 7). DAM conversion occurs in two stages (DAM1 and DAM2) and stage 2 depends on TREM2/TYROBP signaling and presence of APOE^19^.

WAM and DAM2 share many features^18,19^ including expression of phagocytic mRNAs such as CD68 (*Cd68* mRNA), C-type lectin domain containing 7A (*Clec7a* mRNA), and lysozyme (*Lyz2* mRNA), mRNAs associated with lipid metabolisms such as lipoprotein lipase (*Lpl* mRNA) and apolipoprotein E (*Apoe* mRNA), cathepsins B (*Ctsb* mRNA), S (*Ctss* mRNA), and Z (*Ctsz* mRNA), and antigen processing and presentation such as histocompatibility 2, D region locus 1 (*H2-D1* mRNA) or K region locus 1 (*H2-K1* mRNA) (Supp. Data 7). Similarly, activated microglia^18^ (set 2 in Supp. Data 7) share some features with DAM1^19^ (for example, ribosome-related *Rps* and *Rpl* mRNAs and *Aif1* mRNA) (Supp. Data 7). To systematically assess the microglial signatures in our dataset, we calculated the normalized counts of four mRNA sets previously reported in four microglia subtypes in the aging brain by ref. ^18^ (Supp. Data 7). We traced set 1 mRNAs corresponding to WAM/DAM2, set 2 mRNAs corresponding to activated/DAM1 microglia, and sets 3–4 mRNAs corresponding to homeostatic microglia across the three age groups (Fig. 3D). The set 1 WAM/DAM2 mRNAs were dramatically upregulated across all brain regions in the oldest group, but more prominently in the fiber tracts. A modest increase in set 2 activated/DAM1 mRNAs was also noted across the different brain regions (except the fiber tract), suggesting a generally activated state of microglia during aging. Set 3 mRNAs, in general, did not change with age except *Cyba, Fcerg1* and *Tyrobp* mRNAs. Of interest, set 4 homeostatic mRNAs that include a set of cytokines and chemokines involved in homeostatic signaling, were strongly upregulated in the fiber tract (summarized in Fig. 3D). Next, we implemented SPAtial Transcriptomic Analysis (SPATA) to visualize WAM (*n* = 29), DAM1 (*n* = 77), top DAM2 (*n* = 92) signatures across aging. We also included the SenNet^20^ (*n* = 17) focused panel, which includes a gene set commonly upregulated in senescence in the central nervous system. As shown in Fig. 3E, and in congruence with Fig. 3D, WAM and DAM2 mRNA sets were specifically upregulated in the fiber tract region with age. In contrast, the DAM1 signature did not show any prominent changes. Curiously, there was an upregulation of a senescence signature at the fiber tracts suggesting cells localized to this region could be senescent. Notably, 17 out of the original 37 mRNAs in the SenNet panel^20^ could be identified in our processed data.

**Fig. 4:**
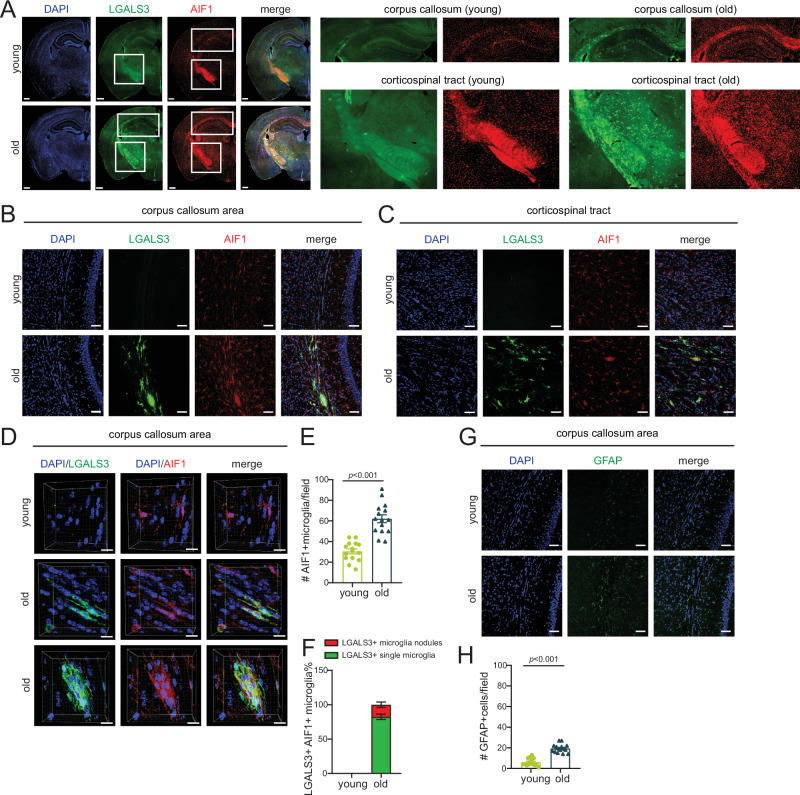
WAM forms nodules at aging fiber tracts. **A** Immunofluorescence microscopy showing the expression of LGALS3 (green) and AIF1 (red) -positive microglia in mouse hemisphere sections with nuclear staining with DAPI (blue). Scale bars are 500 µm. The white insets are magnified on the right and show the corpus callosum and corticospinal tracts in the young and old brain. **B** Confocal images showing expression of LGALS3 (green) and AIF1 (red) -positive microglia in the corpus callosum area. **C** Same as (**B**) except showing corticospinal tract area. For (**B**, **C**), scale bars are 50 µm. **D** Clipped 3D images showing LGALS3 (green) and AIF1 (red) -positive microglia in the corpus callosum of young and old mice. Scale bars are 15 µm. **E** Bar plot showing quantification of AIF-positive microglia in five fields from corpus callosum and corticospinal tract region per mouse (*n* = 3 mice per group; average age, young = 14.67 weeks, old = 85.67 weeks). **F** Bar plot showing the fraction of LGALS3 and AIF1 double positive microglia nodules (18.2%) and LGALS3 and AIF1 single positive microglia (81.8%) quantified from the same fields as (**E**). Data were summarized as mean ± standard error of the mean fraction value of 15 fields of three mice. **G** Confocal images showing expression of GFAP (green) in the corpus callosum. Scale bars are 50 µm. **H** Bar plot showing quantification of GFAP-positive cells from five fields in the corpus callosum and corticospinal tract area per mouse (*n* = 3 mice per group; average age, young = 14.67 weeks, old = 85.67 weeks. For (**E**, **H**), data were summarized as mean ± standard error of the mean cell number from 15 fields of three mice. *p* values are reported from an unpaired two-tailed *t*-test with Welch’s correction comparing old vs young. Source data are provided as a Source Data file.

To validate our spatial transcriptomic findings at the protein level, we performed immunofluorescence assays in young and old mouse brains. In hemisphere sections, we observed strong staining for AIF1 and LGALS3 at the corpus callosum and corticospinal tract area of old murine brains (Fig. 4A, note corresponding DAR images in Supp. Fig. 4G, R). This positive signal was confirmed by zooming in to the corpus callosum or corticospinal tract areas where AIF1 and particularly LGALS3 showed strong age-enrichment (Fig. 4B–E). LGALS3-AIF1-positive microglia assembled in nodular structures (clusters of 3–5) as previously reported for WAM^18^ and shown in the 3D images in Fig. 4D and quantified in 4F. The activated astrocyte marker, GFAP, was also evident in the corpus callosum area of old brains (Fig. 4G, H).

Overall, the increase of inflammatory mRNAs/proteins in the fiber tract area with age indicates a response to injury in this region, which has also been reported in disease conditions such as AD, amyotrophic lateral sclerosis (ALS), multiple sclerosis (MS), and Pelizaeus-Merzbacher disease (PMD)^18,19,21^.

### Evidence of loss of structural integrity in the aging fiber tracts

**Fig. 5:**
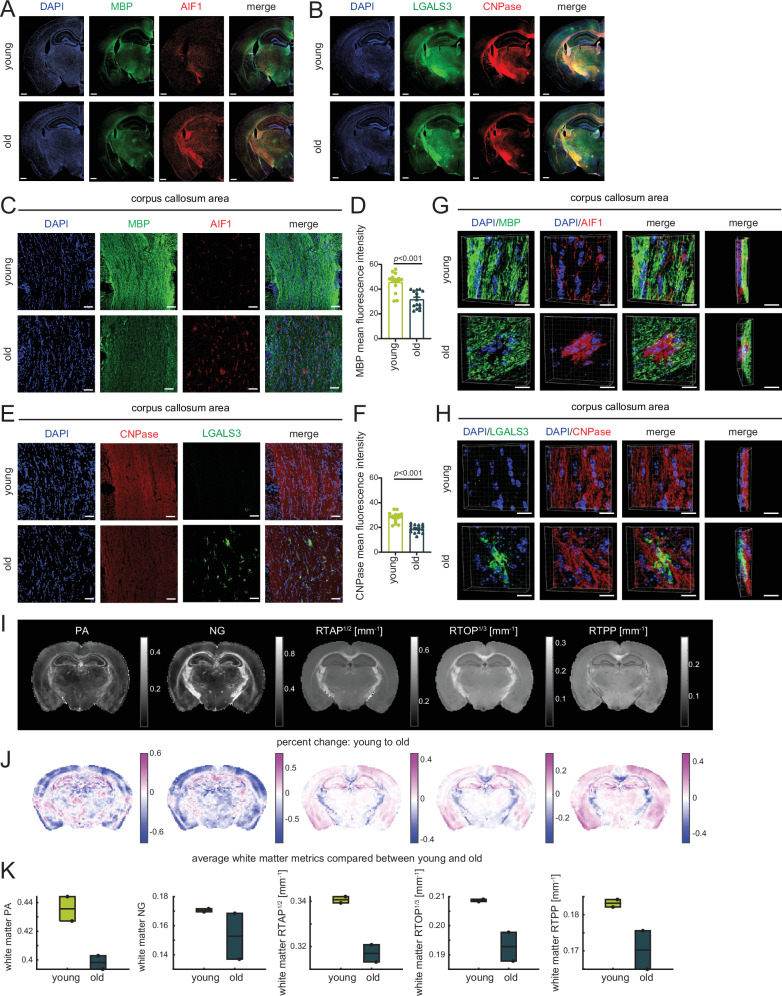
Evidence of loss of structural integrity in the aging fiber tracts. **A** Immunofluorescence microscopy of MBP (green) and AIF1 (red) -positive microglia in mouse hemisphere sections with nuclear staining with DAPI (blue). Scale bars are 500 µm. **B** Immunofluorescence microscopy of LGALS3 (green) and CNPase (red) proteins in the mouse brain. Scale bars are 500 µm. **C** Confocal images of MBP (green) and AIF1 (red) in the corpus callosum area. Scale bars are 50 µm. **D** Bar plot showing quantification of mean fluorescence intensity mean of MBP from five fields in the corpus callosum and corticospinal tract areas per mouse (*n* = 3 mice per group; average age, young = 14.67 weeks, old = 85.67 weeks). **E** Confocal images of LGALS3 (green) and CNPase (red) in the corpus callosum area. Scale bars are 50 µm. **F** Same as (**D**) except quantification from CNPase signal. For (**D**, **F**), data were summarized as mean ± standard error of the mean fluorescence intensity from 15 fields of three mice. *p* values are reported from an unpaired two-tailed *t*-test with Welch’s correction comparing old vs young mice. **G** Clipped 3D images of MBP (green) and AIF1 (red) -positive microglia in the corpus callosum area. The merged images show co-localization of AIF-positive microglia and MBP-labeled myelin at different angles. Scale bars are 15 µm. **H** Clipped 3D images of LGALS3 (green) and CNPase (red) in the corpus callosum area. The merged images show the co-localization of LGALS3+ microglia and CNPase-labeled myelin in the old sections at different angles. Scale bars are 15 µm. **I** Propagator anisotropy (PA), non-Gaussianity (NG), return-to-axis probability (RTAP), return-to-origin probability (RTOP), and return-to-plane probability (RTPP) maps in a representative young sample. **J** Percent change maps of dMRI metrics between group average young and old mice. **K** Box plot showing quantification of white matter PA, NG, RTAP, RTOP, and RTPP in young and old mice. The boxes are bounded by the 25th and 75th percentile values, with the median represented as the bar in the middle. For (**J**, **K**) *n* = 2 biological replicates per group. Source data are provided as a Source Data file.

One manifestation of white matter injury is demyelination. Given previous reports of demyelination events with aging^22^, and the increase with age of the highly phagocytic WAM/DAM2 population engulfing degraded myelin^18^, we looked for evidence of demyelination in our data. We found reduced staining of two myelin-related proteins, myelin basic protein and 2’,3’-cyclic nucleotide 3’-phosphodiesterase (MBP and CNPase, respectively) with age. The decrease in signal for these proteins was evident in hemisphere sections (Fig. 5A, B), and the corpus callosum area (Fig. 5C–F). In 3D images, AIF1- and LGALS3-positive microglia formed large nodules and were in direct opposition to the myelin-rich areas of the old brains (Fig. 5G, H).

We next used diffusion magnetic resonance imaging (dMRI) to investigate microstructural changes in the fiber tract regions with age (Fig. 5I–K). We applied the mean apparent propagator (MAP) model^23^ to ex vivo dMRI data obtained from young and old mice (*n* = 2 biological replicates per group). MAP-MRI explicitly measures the diffusion propagators (i.e., the probability density function of 3D net displacements of diffusing water molecules) in each voxel and can, therefore, capture arbitrary fiber configurations, and in particular, both normal^24^ and abnormal^25^ age-related changes. The zero-displacement parameter maps, return-to-origin probability (RTOP), return-to-axis probability (RTAP), and return-to-plane probability (RTPP), which are all inversely proportional to the spatial dimensions within the microstructure were obtained. We also mapped the non-Gaussianity (NG), which quantifies the dissimilarity between the full propagator and its Gaussian component and reflects the deviation from a simple tensor model, and the propagator anisotropy (PA), which quantifies the directional dependence of the diffusion process. These maps are shown in Fig. 5I from a representative young sample. The Turone atlas (https://www.nitrc.org/docman/?group_id=1411) was used as a common template for registration. Percent change maps of dMRI metrics between group average young and old mice are shown in Fig. 5J, indicating decreases with age in the major white matter tracts, in agreement with our implication of the fiber tract injury with age and in vivo human findings^24^. Quantitative comparisons of dMRI parameters averaged across the whole-brain white matter between the young and old groups are shown in Fig. 5K. Our collective imaging data thus confirms the loss of white matter integrity in the aged brain.

### Innate myeloid immune cells are increased in the old mouse brain

ST can be used to infer cell types from complex mixtures, such as a Visium barcode spot that contains 5–25 cells, by comparing to a reference. We implemented the computational algorithm robust cell-type decomposition (RCTD)^26^, which uses a supervised learning approach to deconvolute cell types while correcting for platform differences. We used as a reference a recently published single-cell dataset of the aging mouse brain from ref. ^27^ containing 25 distinct cell-type signatures. Six cell types were significantly altered with age (Supp. Fig. 6A, B), primary among which were astrocyte restricted precursors (ARPs) which increased with age in the fiber tract. The presence of ARPs in the fiber tract area was interesting and suggests that they co-occur with phagocytic WAM and maybe actively targeting the myelin sheath. Mature neurons showed a trend of decrease with age in the cortical and hippocampal area (Supp. Fig. 6A), although it did not reach statistical significance (Supp. Fig. 6B). Oligodendrocytes showed a trend of increase with age in the fiber tract area (Supp. Fig. 6A) but also did not reach statistical significance (Supp. Fig. 6B).

Neurons, astrocytes, and oligodendrocytes are some of the most abundant cell types in the brain and, therefore, could be visualized easily in the brain sections (Supp. Fig. 6A). Other cell types (not evident by mapping) could be quantified from their calculated RCTD normalized weights and compared across samples. We noted that innate immune myeloid cells (microglia, monocytes, dendritic cells, macrophages, and neutrophils) were selectively over-represented in the aged brain (Supp. Fig. 6B). As a validation, we tested the brain for the presence of neutrophils in both male and female mice (*n* = 5 biological replicates per group). Neutrophils are short-lived cells (lifespan of hours), otherwise found only in peripheral blood by flow cytometry. Importantly, we perfused the animals with PBS to remove any contaminant blood prior to brain harvest. Our data revealed increased numbers of neutrophils in the aged brain parenchyma in both males and females, with differences reaching statistical significance in females (Supp. Fig. 6C–E).

Since aging is accompanied by a myeloid bias in peripheral blood^28^, we questioned whether this increase in neutrophils in the brain was perhaps due to a compromise in BBB integrity. There was evidence in our data of reactive astrogliosis, marked by an increase in *Serpina3n* which encodes α-1 anti-chymotrypsin (α1-ACT), a serine protease inhibitor. In fact, *Serpina3n* was one of the strongest upregulated mRNAs in old brains in our dataset (Fig. 2A, B and Figs. S2A, S3, S4C, S7). α1-ACT is a critical regulator of BBB damage^29^ and its increase typically suggests leakiness in the BBB, as has been evidenced in the aging brain and in disease context^30^. Thus, the presence of neutrophils in the brain is likely due to infiltration incurred from BBB damage, as has been noted in AD brains^31^.

Together, our data indicate two nodes of damage in the aging brain: (1) fiber tracts with extensive demyelination and (2) BBB damage and astrogliosis with infiltration of peripheral blood cells.

### Female mouse brains exhibit stronger pro-inflammatory changes

**Fig. 6:**
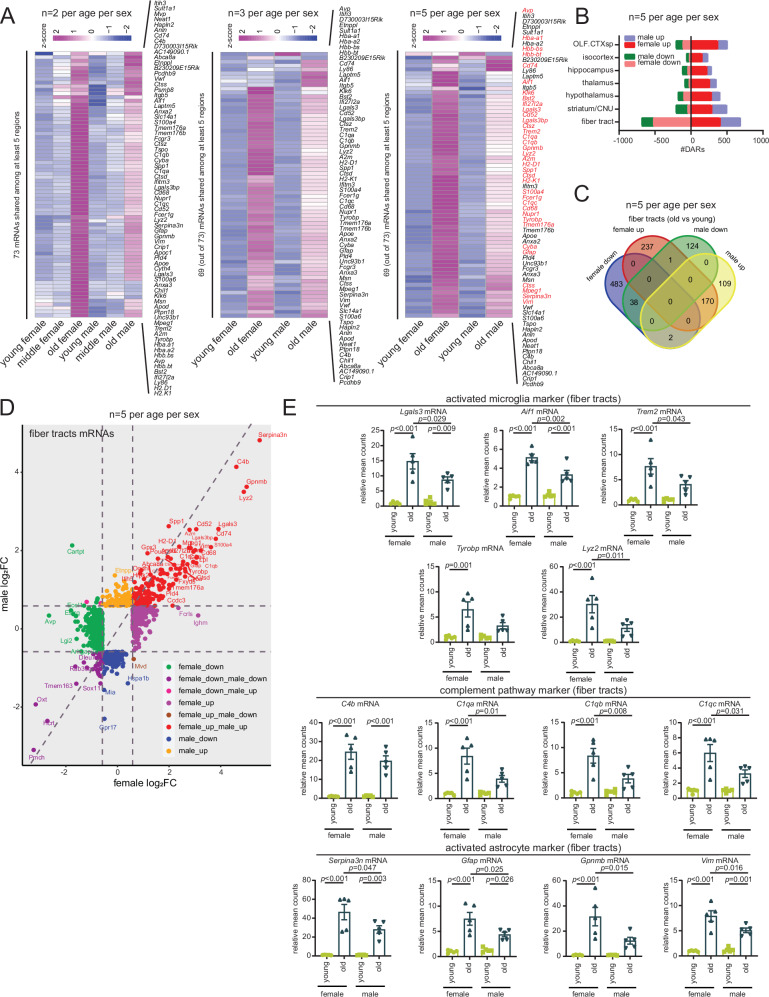
Female mouse brains exhibit stronger pro-inflammatory changes. **A** Heatmap of count mean values for 73 mRNAs shared among at least five regions grouped by age and sex at pseudobulk level (left). Heatmap of count mean values of 69 of the 73 mRNAs detected in the second round of experiments with *n* = 3 biological replicates per age per sex (middle). Heatmap of the count mean values of 69 of the 73 mRNAs with *n* = 5 biological replicate per age per sex. *n* = 37 out 69 DARs significantly higher in old females than old males (FDR <0.05, and FC ≥1.5) are labeled red (right). **B** Bar plot showing the number of age-related DARs in female and male mice split by brain region. The age-related mRNAs were filtered by FDR <0.05 and FC ≥1.5 or FC ≤1.5 in old vs. young. **C** Venn diagram showing overlap of four indicated groups of DARs in old vs. young, male and female mice in fiber tracts. **D** Scatterplot showing significant DARs in old vs. young male and female mice (FDR <0.05, FC ≥1.5 or ≤−1.5) in fiber tracts. **E** Bar plot of activated microglia marker mRNAs *Lgals3*, *Aif1*, *Trem2*, *Tyrobp*, and *Lyz2*; complement pathway marker mRNAs *C4b*, *C1qa*, *C1qb*, and *C1qc*; and activated astrocyte marker mRNAs *Serpina3n, Gfap, Gpnmb*, and *Vim* in fiber tracts of young and old, male and female mice. Data were summarized as mean ± standard error of the relative mean counts. Each dot shows the expression in an individual animal relative to the mean of the young female group, equivalent to *n* = 5 biological replicates per age per sex. *p* values are reported from a one-way ANOVA with Tukey’s multiple comparisons test. Source data are provided as a Source Data file.

The impact of sex on neurodegeneration is a complex and poorly understood topic, however, there are clear differences in incidence rates, clinical manifestations, and progression driven by sex hormones or genes on sex chromosomes. Our primary dataset (Figs. 1–5 and Supp. Figs. 1–5) had representation from both sexes but was not powered to address sex-specific differences (*n* = 2 per age per sex). Nevertheless, when analyzing the 73 (mostly inflammatory) mRNAs commonly upregulated with age across five brain regions (Fig. 2G), it was clear that females showed stronger expression than males (Fig. 6A, left). We next performed a priori power analysis to determine a reasonable sample size required to confidently assess sex differences in ST data. Accordingly, we added three additional biological replicates of young and old mouse brains for a total of *n* = 5 per age per sex. An increase of sample size from *n* = 2 to *n* = 5 led to a power increase from 0.51 to 0.98, considering a difference between means of 3.5 S.D. and an FDR <0.05 for identifying DARs. When analyzing the new data (*n* = 3 per age per sex, Fig. 6A, middle) or combined with our primary dataset (which we call cohort 2, *n* = 5 per age per sex, Fig. 6A, right), we noted stronger transcriptional signatures in females compared to males. These results also confirm the reproducibility of our results. Additionally, in cohort 2, we find *n* = 37 out of 69 DARs were significantly higher in old females than old males (FDR <0.05, and FC ≥1.5, labeled red in Fig. 6A, right). Notably, we re-pooled the young and old libraries from our primary dataset with the three additional replicates and sequenced all libraries together for the combined analysis to minimize batch effects due to sequencing. We noted that females had more DARs compared to males (although there was a significant overlap of upregulated mRNAs) and that the fiber tracts had an overrepresentation of the upregulated DARs compared to other regions (Fig. 6B, C). These pro-inflammatory DARs are shown as a scatterplot in pseudobulk data from all brain regions in Supp. Fig. 7B and specifically for fiber tracts in Fig. 6D. A few select DARs are further graphed to show within-group variations across the 5 animals in Fig. 6E. Together, our results suggest that, at least in mice, the age-related neuroinflammatory response is stronger in females at the transcriptional level.

Regions other than fiber tracts that showed sex-biased expression included (among others) hippocampus, thalamus, and hypothalamus (Supp. Fig. 7C–E and Supp. Data 9). Of mRNAs with sex-specific regulation during aging, the mRNAs encoding chloride intracellular channel 6 (*Clic6* mRNA) and transthyretin (*Ttr* mRNA), both involved in chronic psychogenic stress, are upregulated in female hippocampus with age but downregulated in males (Supp. Fig. 7C). The mRNA encoding cortexin 3 (*Ctxn3* mRNA), is selectively upregulated in the thalamus of aged male mice but downregulated in females (Supp. Fig. 7D). Similarly, the precerebellin mRNA (*Cbln1* mRNA), important for synapse integrity and synaptic plasticity was downregulated in females but upregulated in males in the thalamus and fiber tracts (Fig. 6D and Supp. Fig. 7D). In contrast, antidiuretic hormone vasopressin (*Avp* mRNA) expression was upregulated in the thalamus in female mice but strongly downregulated in males (Supp. Fig. 7D). In the hypothalamus, the leucine-rich repeat containing 17 mRNA (*Lrrc17* mRNA) was upregulated in male mice but downregulated in females (Supp. Fig. 7E).

Overall, there are many sex-specific differences in the transcriptomic profiles of male and female aging brains, with the latter showing stronger inflammatory features.

### Machine learning prediction of epigenetic drivers of gene expression in the aging mouse brain

**Fig. 7:**
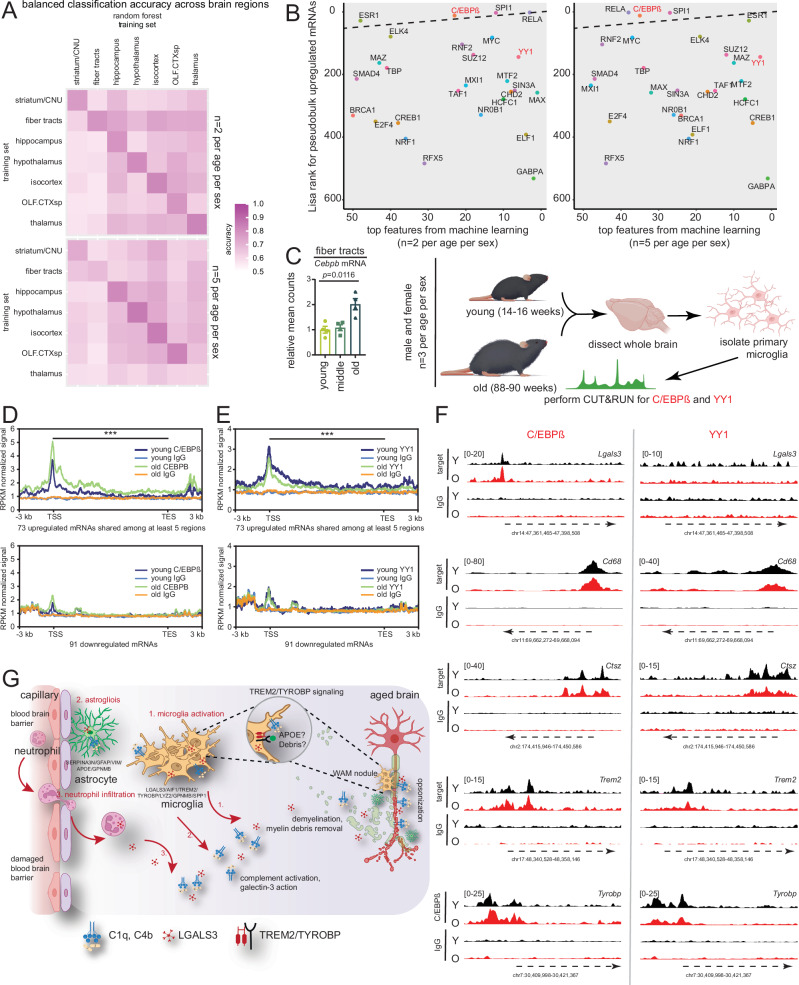
Machine learning prediction of epigenetic drivers of gene expression in the aging mouse brain. **A** Heatmap showing the balanced classification accuracy of random forest models for cohort 1 (top) and cohort 2 (bottom) over three classes (up/down/constant) across seven brain regions. The final prediction was defined as up or down if at least 75% of the models agreed, as previously described^33^. For cross-region validation, the entire data of the tested region was used. The color scale shows the accuracy values. **B** Scatterplot of top 50 features obtained from machine learning on cohort 1 (left) or cohort 2 (right) and Lisa ranks of predicted TFs from upregulated DARs in pseudobulk data. C/EBPβ and YY1, labeled in red, were chosen for testing. **C** Bar plot of *Cebpb* mRNA in fiber tracts of young, middle, and old mice. Each dot shows the average expression of the two technical replicates for each mouse relative to mean of young group, equivalent to *n* = 4 biological replicates per group. Data were summarized as mean ± standard error of the relative mean counts. **p < 0.01, from an unpaired two-tailed *t*-test with Welch’s correction comparing middle vs. young and old vs. young (left). Schematic showing the study design of CUT&RUN experiments in primary microglia isolated from young and old mouse brains (right). **D** Metaplot showing binding of C/EBPβ near TSSs of 73 genes upregulated during aging (Fig. 2D, G) and shared across five regions (top) or a background set of 91 genes that are downregulated with age also from Fig. 2D (bottom). **E** Same as (**D**), except for YY1. For (**D**, **E**), ***p < 0.001 from an unpaired two-tailed *t*-test with Welch’s correction comparing AUC values across the gene body in old vs young. **F** Representative genome browser tracks showing binding of C/EBPβ (left) and YY1 (right) at key upregulated genes. **G** Comprehensive model derived from this study showing BBB damage and demyelination at fiber tracts with consequential microglia activation (WAM/DAM2 signature), astrogliosis, and neutrophil infiltration. Illustration credit for parts of (**C**) goes to Endosymbiont GmbH, and BioRender. Sen, P. (2025) https://BioRender.com/m70w321. Source data are provided as a Source Data file.

Gene expression is tightly regulated by the activity of transcription factors (TFs), proteins that bind to specific DNA sequences located in upstream promoter regions of genes. Therefore, we sought to build a predictive model to identify key TFs most likely to regulate gene expression changes in the aging brain (see Methods). We did this separately for each of the seven brain regions identified in our data (Fig. 1C). The two inputs in this process were (1) ChIP-seq data for the TFs of interest and (2) transcriptomics data for the target genes in each region. For the first input data from ref. ^32^, was a subset for TF binding, and codified into a binary format representing the presence or absence of target gene binding for each TF. For the second input, we used our ST data from this study (cohort 1 and 2 separately) categorized as up if the FC >1.2, down if <−1.2. For cohort 1 an FDR <0.05 was used whereas for cohort 2 FDR <0.1 was used. Any mRNA not passing the FDR or FC filter was categorized as constant. The integrated inputs allowed for the creation of a matrix (Supp. Data 10)^33^ that represented the binding of the TFs to the regulatory regions of the target genes. We then trained random forest models to predict whether an mRNA is upregulated or downregulated in a given brain region using the TF binding profile as model features. Our results indicate that training on one brain region best predicts expression within that region (Fig. 7A, cohort 1 on top and cohort 2 on bottom), but also that trained models can generalize to other regions, arguably with lower accuracy, indicating shared features and regulatory pathways between brain regions.

To identify the most informative features (most influential TFs), we ranked the TFs according to their impact on prediction performance when the corresponding TF was removed from the training set (see Methods). The top 50 most important proteins were chosen for further analysis from cohort 1 and 2 with 35 proteins shared between the two. To further increase our predictive power, we performed Lisa analysis^34^, a method that leverages a comprehensive database of ChIP-seq and DNase-seq data (CistromeDB) to identify TFs and other chromatin regulators perturbing a user-provided gene set (in our case, pseudobulk upregulated DARs). Figure 7B shows a scatterplot of chromatin regulators based on their rank in Lisa and machine learning. We selected two TFs, CCAAT/enhancer binding protein β (C/EBPβ) and Yin Yang 1 (YY1), both known to mediate inflammatory gene expression in myeloid cells^35,36^, for further validation. Additionally, *Cebpb* was among the unique mRNAs upregulated with age in the fiber tract (Fig. 7C, left and Supp. Data 5).

We isolated primary microglia from young and old brains (*n* = 3 biological replicates per age per sex) using a bead-based method and performed Cleavage Under Targets and Release Using Nuclease (CUT&RUN)^37^ with C/EBPβ or YY1 antibodies (Fig. 7C, right). Control reactions were incubated with IgG antibodies. Binding patterns of both TFs revealed strong enrichments near TSSs of the 73 upregulated genes shared across five regions from Fig. 2D, G (Fig. 7D, E, top panels). C/EBPβ showed a statistically significant increase in binding with age, while YY1 showed a slight (but significant) decrease, although the effect sizes were small. In contrast, we observed much weaker binding at the 91 downregulated genes from Fig. 2D (Fig. 7D, E, bottom panels), which was not statistically different between the age groups. Some representative browser shots of C/EBPβ and YY1 binding are shown in Fig. 7F (from the gene set in Fig. 7D, E top). While most of the genes were co-bound with C/EBPβ and YY1, some, such as *Lgals3*, were exclusively bound by C/EBPβ (Fig. 7F, top). More importantly, we noted that both TFs were bound even in the young state, suggesting that inflammatory genes are primed for expression by the pre-binding of critical TFs. Further work is needed to elucidate the role of coactivator recruitment by these TFs during brain aging.

## Discussion

In this study, we performed systematic, spatiotemporal mapping of the aging mouse brain spanning three age groups (young, middle, and old), both sexes (male and female), and two technical replicates per animal (A and B) for a total of 24 high-quality, high-depth, reproducible datasets (Fig. 1 and Supp. Fig. 1). This initial cohort data covered a total of 67,998 spatial spots, with an average tissue coverage of 2833, ~56 K mean reads, and ~5219 median genes per spot. The average coverage of 2833 out of 5000 spots within a Visium capture area represents ~56% and aligns with Image J estimates of tissue coverage for a single coronal hemisphere section. These parameters were comparable in cohort 2 where we increased the sample size for estimation of sex differences (total *n* = 5 per age per sex, Supp. Data 2) for a total of 36 Visium libraries combining both cohorts. This benchmarking process lent confidence to our sample preparation step.

We found that it was possible to develop a detailed whole-brain spatial annotation based purely on gene expression signatures (Figs. 1B–F, 2C, top). Using a pseudobulk approach first, we identified a strong immune signature in the aging brain (Fig. 2 and Supp. Figs. 2, 5) as has been reported in numerous previous studies (reviewed in ref. ^9^). To unambiguously discern DARs that emerge due to age, we incorporated a middle-age group as a design feature in our study in this initial cohort. Our spatial data was thus able to derive all possible differential gene expression trajectories across the lifespan (Supp. Fig. 3). We found 37 DARs were upregulated from middle-age onwards (Supp. Fig. 3, clusters 1–2) while 112 DARs in old age (Supp. Fig. 3, cluster 4). Downregulated DARs were fewer with 23 DARs that declined from middle-age (Supp. Fig. 3, clusters 9–10). Additionally, we found smaller DAR sets that were exclusively up- or down-regulated in middle-age (Supp. Fig. 3, clusters 5–7 (up), *n* = 22 and clusters 3 and 8 (down), *n* = 7). Importantly, we found that the age-related immune signature (primarily cluster 4) was most prominent in the oldest group (average age 126 weeks) while being only modestly upregulated in the middle-age group (average age 57.5 weeks) (Fig. 2 and Supp. Figs. 2–7), suggesting that inflammatory changes in the brain occur late in life. Furthermore, we found that a smaller group of mRNAs related to synaptic function was downregulated with age (Fig. 2 and Supp. Figs. 2, 3, 5), suggesting a loss of brain connectivity. We speculate the reason we find more upregulated than downregulated DARs is because neuroinflammation is an active response, driven by TFs (Fig. 7). Additionally, we find that at least two tested TFs, C/EBPβ and YY1, are already bound to TSS regions of upregulated DARs in the young, suggesting these genes are primed to launch a robust response. In turn, this suggests that glia-mediated clearance mechanisms are programmatically activated in the aging brain. In contrast, the weaker downregulation of synaptic genes is indicative of secondary degenerative processes in the neurons.

Our dataset thus presents a systematic, reproducible view of age-related neuroinflammation and reflects a snapshot of processes likely to be physiologically relevant. Most single-cell investigations of the aging or diseased brain involve making single-cell suspensions from tissue homogenates, with the loss of relevant cellular connections through ligand-receptor pathways. In fact, a previous seminal study^38^ reported that mature microglia rapidly lose their identity after isolation but regain it after engraftment back into the central nervous system environment, suggesting that continuous niche input is required to maintain its structure and function. Other studies have used microglia and astrocytic cultures in a serum-containing medium, which dramatically alter expression profiles and phagocytic activity, perhaps mitigating differences due to age^38,39^. Our data, in contrast, overcomes these challenges with direct expression profiling in situ and, therefore, shows many statistically significant changes with robust fold changes. Notably, we find some mRNAs not reported in prior single-cell studies of the aging brain^27,40,41^. For example, *Gpnmb*, an mRNA upregulated with age in our dataset, is a transmembrane senescence marker recently reported in the context of atherosclerosis^42^ and found to be elevated in microglia in AD mouse models and human patients, along with TREM2 and APOE^43^. CCAAT/enhancer binding protein delta (*Cebpd* mRNA), also upregulated in our dataset, is an innate immune player implicated in AD^44^. Other examples are protein tyrosine phosphatase non-receptor type 18 (*Ptpn18* mRNA), coiled-coil domain containing 3 (*Ccdc3* mRNA), and Strawberry notch homolog 2 (*Sbno2* mRNA).

Interestingly, differential mRNA abundance analysis on each individual brain region identified from the spatial profiles highlighted the fiber tract as a particularly sensitive area, showing not only the largest number of DARs but also the largest FC in expression (Fig. 2 and Supp. Fig. 2). Aging fiber tracts have been implicated as a vulnerable region in an independent spatial study while this manuscript was in preparation^45^ and were previously identified as a degenerative center in MRI measures of myelin content and axonal density^46–50^. Our dMRI results showed significant age-related decreases in white matter for the PA and RTAP indices (Fig. 5I–K), suggesting reduced fiber orientational coherence, and an increase in microscopic water displacement, respectively, with age. These findings support the microstructural scenario of axonal degradation with age, leading to increased extracellular volume, in which water diffuses almost freely with little anisotropy but with a diffusivity similar to that of cerebrospinal fluid (CSF). Additionally, a recent publication using multiplexed error-robust fluorescent in situ hybridization (MERFISH), combined with single-nucleus RNA sequencing (snRNA-seq), identified the white matter as a hotspot for age-related glial and immune cell activation^51^. Thus, our data, together with results from independent studies across diverse platforms, pinpoints fiber tracts as focal points of brain aging.

Why might the white matter be a hotspot for inflammation? We believe that white matter is an early target during aging due to its unique macromolecular composition, vascularity, cellular composition, and structure. The white matter is rich in lipids, forming lipid peroxide products upon oxidation that are highly damaging^52^. The white matter is less vascularized than gray matter^53,54^ making it also susceptible to hypoxic damage. It is also possible that oligodendrocytes or oligodendrocyte progenitor cells (OPCs) in the fiber tract area may become senescent, releasing senescence-associated secretory phenotype (SASP) factors, and attracting microglia. Although not directly tested, we find a modest senescence signature in the fiber tract area (Fig. 3E). Additionally, senescent OPCs have been found around Aβ plaques in AD mouse models and human brains. Finally, complement proteins, APOE and LGALS3, can serve as opsonins^55,56^, coating the white matter tracts for microglia targeting. Our snapshot view may be capturing this ongoing event. Relatedly, we find many more upregulated DARs in this area than downregulated DARs, with fewer GO hits for downregulated mRNAs (Supp. Fig. 5). It is possible that when it comes to upregulation, there is a signal amplification and spreading process at fiber tracts. For example, it is possible that senescent oligodendrocytes induce bystander senescence which releases more SASP. Or that there is a high concentration of myelin debris near fiber tracts that can cause chronic microglial activation, which in turn activates other nearby microglia. Chronic exposures are also associated with microglial priming^57^, an exaggerated response to a secondary stimulus. In contrast, downregulated DARs, mostly related to synaptic function, are secondary degenerative effects that are not well-controlled responses and do not have an amplification component.

The mRNA signature at fiber tracts (and to a lesser extent in the hippocampus, thalamus, and hypothalamus) had three major indications: microglia activation, astrogliosis, and innate immune myeloid cell infiltration (summarized in Fig. 7G). Accumulation and activation of innate immune cells in heavily myelinated fiber tracts in the old brain underscore the involvement of immune cells in demyelination/remyelination or myelin debris removal during natural aging. Previous literature has reported that aging and neurodegeneration activate distinct microglia subtypes: WAM^18^ and DAM^19^. In mouse models of AD, the WAM signature appears early, followed by gradual upregulation of DAM1 and DAM2^18^. The high phagocytic activity of DAM2 triggered in the disease context serves to clear degraded myelin and is said to be protective. We investigated WAM and DAM expression signatures and could identify both as being activated in our dataset (Fig. 4). In particular, *Trem2, Tyrobp*, *Lgals3* mRNA, and early complement pathway mRNAs *C1q* and *C4b* were remarkably upregulated in microglia in old white matter, implicating their involvement in aging-related fiber tract pathophysiology.

TREM2 promotes myelin debris removal by microglia in naturally aging brains, highlighting its neuroprotective role^18^. However, TREM2 was also shown to convert microglia phenotype from homeostatic to neurodegenerative, and ablation of TREM2 in AD mice resulted in suppression of inflammatory reaction^58^. Furthermore, in humans, *TREM2* variants posea risk for ADRD^59^. Whether TREM2/TYROBP signaling in the microglia of old brains, particularly in fiber tracts, serves a neuroprotective or neurodegenerative role, or both, remains to be determined^41^.

Similarly, elevated LGALS3, a WAM marker, was largely shown to be detrimental in the brain, with an implied protective function depending on the context of the surrounding microenvironment^60^. Activated but not homeostatic microglia express LGALS3^58^. LGALS3 was increased in neurodegenerative microglia in AD patients, and AD and MS mouse models, and ablation of LGALS3 suppressed inflammatory response and alleviated AD or MS phenotypes in mice^58,61,62^, emphasizing neurodegenerative effects of LGALS3 in the context of AD or MS. Furthermore, the proinflammatory action of LGALS3 was shown to be mediated by TREM2/TYROBP^61^ or TLR4^63^ in the brain, where LGALS3 functions as an endogenous paracrine ligand of these receptors. However, LGALS3 was required for M2 polarization in peripheral lung or peritoneal macrophages^64^, suggesting its protective anti-inflammatory potential in macrophages. We found that *Lgals3* expression is strikingly upregulated in microglia, and about 20% of LGALS3-positive microglia form nodules that physically interact with myelin in fiber tracts in the old brain (Figs. 3–5). We infer that LGALS3 and TREM2 are involved in the deterioration of white matter myelin sheath during natural brain aging separately or in combination.

C1q was previously reported to increase in the aging brain^65^, and we found evidence of early complement activation, with the expression of *C1q* and *C4b* mRNAs. The C1q-induced classical complement pathway plays a critical role in synaptic pruning^66^, and C1q-ablated mice exhibit improved synaptic plasticity and alleviation of cognitive and memory decline during aging^65^. In MS, early complement pathway proteins were shown to be increased around progressive white matter lesions^67,68^, suggesting an involvement of the complement pathway in MS pathophysiology, i.e., demyelination and myelin debris removal^69^. Consistently, the complement proteins, APOE and LGALS3 are known to opsonize the myelin sheath in white matter to promote recognition and clustering of microglia in this region for targeted phagocytosis^56^. Collectively, activated microglia and the classical complement pathway likely contribute to myelin degeneration in white matter during natural brain aging.

Another important feature evident in our data was reactive astrogliosis, which suggests a compromise of the extracellular matrix and BBB integrity, although this was not directly tested in this study^29^. Loss of BBB integrity with age facilitates infiltration of peripheral blood cells into the brain parenchyma, relegating the immune-privileged status of the brain. Using computation (RCTD) and experimentation (flow cytometry), we detected an increased myeloid cell signature and infiltration of neutrophils in the aged brain (Supp. Fig. 6). Together with activation of resident immune cells like microglia and astrocytes, infiltrating cells can aggravate inflammation, nerve degeneration including demyelination and cognitive decline with age^70^.

Our dataset underscores the importance of studying both sexes in aging and demonstrates that neuroinflammation is highly sex-dimorphic, with females showing a stronger response (Fig. 6). A sex-specific analysis was empowered by our inclusion of a second cohort of three additional biological replicates per sex group in the young and old categories (total *n* = 5 biological replicates per age per sex). We further validated our sex dimorphic data by flow cytometry in an independent set of animals (*n* = 5 biological replicates per group), which shows that aged female mice have greater numbers of infiltrating neutrophils in the brain parenchyma (Supp. Fig. 6). Notably, recent studies that identified microglia activation and astrogliosis by Visium profiled either only male brains^45^ or by MERFISH and snRNA-seq only female brains^40,51^. Our data is concordant with previous reports in humans that show greater immune activation in the hippocampus and cortex of old female brains^71^. Aged women also show higher overall prevalence of all-cause dementia^72^. The consistent sexual dimorphism observed in these different studies likely indicates a higher risk in females for age-related loss of brain function.

In addition to showing strong female bias, our dataset has several distinct advantages over recently published papers. Our sample numbers (*n* = 5 biological replicates per age per sex, or *n* = 10 per age) exceed published single-cell or spatial datasets in aging mouse brains^27,40,45,51^ (*n* = 2–8 biological replicates per group) and include a geriatric group where we note the strongest inflammatory signatures. Our use of coronal sections (similar to ref. ^45^) allowed us to interrogate seven distinct anatomical regions, while ref. ^51^ focused on the frontal cortex and striatum. A major strength in our study is the additional validations using multiple orthogonal techniques (Figs. 4, 5, 7 and Supp. Fig. 6). A key outcome of this validation was the identification of two TFs, C/EBPβ, and YY1 that bind in the TSS regions of neuroinflammatory genes.

Despite the above advantages, our use of coronal sections at defined anterior-posterior positions in the brain has limited our ability to query all possible brain regions. Furthermore, we have used a fairly gross resolution for clustering. A technical limitation is that the Visium slides used here do not reach single-cell resolution, having ~5–25 cells per spot from a 16 µm section. As higher-resolution discovery and validation platforms are being developed, we will be able to pursue finer-grained dissection of substructures in the brain.

In summary, we identified striking global transcriptomic alterations in seven anatomically distinct brain regions during normal aging, with the core findings of prominent changes in white matter fiber tracts, demonstrating widespread and sex-dimorphic patterns of increased inflammatory gene signatures and decreased synaptic plasticity, likely providing valuable resources for future mechanistic studies of normal brain aging and possibly insights into the pathogenesis of Alzheimer’s Disease and related Dementias (ADRD).

## Methods

### Animals

This study was approved by the Animal Care and Use Committee of the NIA in Baltimore, MD, under Animal Study Protocol number 481-LGG-2025. Young, middle-aged, and old inbred C57BL6/JN mice of both sexes were acquired from the NIA-aged rodent colony (https://ros.nia.nih.gov/) and housed in rooms that were maintained at 22.2 ± 1 °C and 30–70% humidity. Routine tests are performed to ensure that mice are pathogen-free and sentinel cages are maintained and tested according to the American Association for Accreditation of Laboratory Animal Care (AAALAC) criteria. The age and sex information are available in Supp. Data 1.

### Brain dissection, freeze-embedding, and sectioning

For ST, mice were euthanized following the 2013 AVMA Euthanasia guidelines by carbon dioxide asphyxiation and then quickly decapitated. Fresh brains were extracted from the mouse skull and the cerebral hemispheres are partially separated from each other along the interhemispheric fissure (deep groove) of the falx cerebri. The obtained tissue samples were covered in room temperature optimal cutting temperature (OCT, Tissue-Tek) followed by simultaneous freezing and embedding in a bath of isopentane and liquid nitrogen to prevent RNA degradation and avoid morphological damage. The frozen OCT-embedded tissue block was stored in a sealed container at −80 °C until ready for cryo-sectioning. Coronal sections of thickness 16 µm were cut using a CM3050S cryostat (Leica). We focused on capturing sections with the hippocampus, as this is a region implicated in age-related cognitive decline. Consequently, we chose positions 277–293 (an ~400 µm window) from the P56 coronal Allen Mouse Brain Atlas^14^ across different animals. We tried to be as consistent as practically possible to select the same region focused on the middle of the hippocampal structure along the dorsoventral axis. Tissue sections were placed within the frames of the capture areas on a Visium spatial tissue optimization slide (capture area 8 × 8 mm, surrounded by an etched frame, 10X Genomics, PN3000394) or Visium spatial gene expression slides (capture area 6.5 × 6.5 mm, surrounded by a fiducial frame for a total area of 8 × 8 mm, 10X Genomics, PN2000233). The slides were stored individually in a sealed container at −80 °C until the next step. The fresh frozen protocol was followed for ST.

### Fixation, H&E staining, and imaging of tissue sections

The slides containing tissue sections were removed from −80 °C and transported to lab space on dry ice in a sealed container. The thermocycler adapter (10X Genomics, PN3000380) was placed on a thermomixer C (Eppendorf) set at 37 °C for 5 min. The slide was incubated on the thermocycler adapter with the active surface facing up for 1 min at 37 °C and then immersed in prechilled methanol (Sigma-Aldrich) for 30 min at −20 °C. After fixation, the slide was uniformly covered in isopropanol (Sigma-Aldrich) for 1 min at room temperature and air dried (not exceeding 10 min). After drying, the slide was stained in Mayer’s hematoxylin (Dako, S3309) for 7 min at room temperature, followed by 35 washes in water. Subsequently, the slide was incubated in a bluing buffer (Dako, CS702) for 2 min at room temperature, followed by five washes in water. The slide was then stained in a prepared Eosin mix for 1 min at room temperature (100 µl Eosin (Dako, CS701) and 900 µl 0.45 M Tris-acetic acid buffer, pH 6.0) followed by 15 washes in water. The tissue was air dried until opaque followed by incubation on the thermocycler adapter for 5 min at 37 °C. The slide was imaged on the all-in-one fluorescence microscope (Keyence, BZ-X710) at the desired magnification using brightfield imaging settings. The spatial gene expression imaging guideline technical note (10X Genomics, CG000241) was consulted for reference.

### Optimization of tissue permeabilization times

To optimize permeabilization time for mouse brain tissue, the Visium spatial tissue optimization slide was placed in the slide cassette (10X Genomics, PN3000406). The tissue sections on the slide were incubated with the permeabilization enzyme (10X Genomics, PN2000214) for a time course of 30, 24, 18, 12, 6, and 3 min to identify an optimal tissue permeabilization time for further use in the visium spatial gene expression. About 70 µl permeabilization enzyme was added to the first well, and then the slide cassette was placed on the thermocycler adapter at 37 °C. After 6 mins, 70 µl permeabilization enzyme was added to the second well and the process was repeated for the other wells until the shortest incubation time 3 min. After time course completion, all wells were washed with 100 µl 0.1X SSC buffer (15 mM NaCl, 1.5 mM sodium citrate, pH 7.0). Subsequently, the reverse transcription (RT) master mix containing RT reagents and fluorescently labeled nucleotides (10X Genomics, PN1000192) was added on top of the tissue sections to make fluorescently labeled cDNA. After cDNA synthesis, tissue sections were removed with the tissue removal mix (10X Genomics, PN1000191) and then washed with 0.2X SSC and 0.1X SSC buffer. After removing all the tissues on the slide, all capture areas were imaged by the all-in-one fluorescence microscope (Keyence, BZ-X710) at the desired magnification using fluorescence imaging settings. The spatial gene expression imaging guideline technical note (10X Genomics, CG000241) was consulted for reference. In our studies, 30 min was identified as the optimal permeabilization time for 16 µm sections from pilot studies using both young and old brains. Although there was a saturation of fluorescence signal from 18–30 min, we decided to use 30 min for all age groups to reduce any technical variability due to different permeabilization times and ensure all regions were well permeabilized. We carefully analyzed the size distribution of extracted cDNAs for indications of degradation. Our analyses revealed no significant changes (Supp. Fig. 1B, top panel). Additionally, we performed downstream count normalization, which tackles any global differences in RNA extraction (Supp. Fig. 1D).

### On-slide tissue permeabilization, cDNA synthesis, and probe release

The Visium gene expression slide was retrieved post-fixation and H&E staining. About 70 µl permeabilization enzyme (10X Genomics, PN2000214) was added to the fixed and stained sections and the slide was incubated for 30 min at 37 °C. After permeabilization, all wells were washed with 100 µl 0.1X SSC buffer followed by addition of 75 µl RT master mix. After RT, the tissue sections were incubated with 75 µl 0.08 M KOH for 5 min, washed with 100 µl elution buffer (EB, Qiagen), and then treated with 75 µl s strand mix. After the second strand synthesis, the cDNA from each capture area was washed with 100 µl buffer EB and then denatured and diluted with 35 µl 0.08 M KOH and 5 µl Tris (1 M, pH 7.0). cDNA (35 µl) from each well was transferred to a corresponding tube before proceeding to cDNA amplification and library construction.

### cDNA amplification

A 1 µl cDNA sample was used for the estimation of cycles of amplification using the Kapa SYBR fast qPCR master mix (Kapa Biosystems, KK4600) and cDNA primers (10X Genomics, PN2000089). A total of 25 cycles was first performed, and the threshold was set along the exponential phase of the amplification plot at ~25% of the peak fluorescence value. In the end, our estimate determined 17 cycles of cDNA amplification. Spatially barcoded full-length cDNA from all samples were amplified using 17 cycles PCR by adding 65 µl cDNA amplification mix (10X Genomics, PN2000189).

### ST library construction and sequencing

The post-cDNA amplification product was cleaned with SPRIselect (Beckman, Coulter B23318) and eluted in a volume of 40 µl; 1 µl of eluate was run on an Agilent bioanalyzer DNA 1000 chip (Agilent Technologies, 5067-1504) to assess cDNA quality and quantity and 10 µl (25%) of the total amplified cDNA was used as input to perform enzymatic fragmentation, end repair and A-tailing (10X Genomics, PN1000196). A double-sided size selection was then performed with SPRIselect (Beckman, Coulter B23318). TruSeq read2 primer was added via adapter ligation (10X Genomics, PN1000196). P5, P7, i7, and i5 sample indices were added via index PCR with the individual dual index TT set A (10X Genomics, 3000431). The cycle number for index PCR was calculated based on the yield of the 25% carry forward cDNA material, and 15 cycles were used. A double-sided size selection of the post-index PCR sample was performed with SPRIselect (Beckman, Coulter B23318). The purified library (1 µl) was run on an Agilent bioanalyzer DNA 1000 chip (Agilent Technologies, 5067-1504) to assess cDNA quality and quantity. The average fragment size was determined from the bioanalyzer trace and used as the insert size for library quantification. Libraries from individual samples were pooled in equimolar amounts, re-quantified using the NEBNext Quant kit (New England Biolabs), and loaded on the NovaSeq 6000 sequencer (Illumina). For the initial cohort, two runs were performed using NovaSeq S1 100 cycle kits for a total of 4.1 billion paired-end reads. For the second cohort, one NovaSeq S1 100 cycle kit was used for a total of 2 billion paired-end reads.

### Immunofluorescence imaging

Mouse brain coronal sections (30 µm) were cut from an OCT block onto positively charged glass slides using a CM3050S cryostat (Leica). Floating sections were first incubated in permeabilization buffer (0.2% Triton™ X-100 in 1X PBS) for 5 min at room temperature. Antigen retrieval was performed by placing the sections in 1X sodium citrate solution (Thermo Fisher) in an 80 °C bath for 30 min, followed by incubation in blocking buffer (5% normal serum, 0.3% Triton™ X-100 in 1X PBS) for 1 h at room temperature. Sections were incubated with primary antibodies diluted in antibody dilution buffer (1% BSA, 0.3% Triton™ X-100 in 1X PBS) overnight at 4 °C. The sections were then incubated with secondary antibody conjugated to a fluorescent dye for 2 h at room temperature, followed by washes with PBST and staining with 5 µg/ml DAPI for 15 min at room temperature. After a wash with PBS, the sections were mounted with Epredia Lab Vision PermaFluor Aqueous Mounting Medium (Fisher Scientific). Photographs were taken using a Zeiss LSM 710 confocal microscope. The quantification was accomplished from *n* = 3 mice per group; average age, young = 14.67 weeks, old = 85.67 weeks. Five fields from the corpus callosum and corticospinal tract region per mouse were captured using a 20X objective. In each field, the fiber tract area of interest was marked for quantification of myelin. Alternatively, IBA1+ or LGALS3+ microglia were labeled in each field and cell numbers/intensities were quantified using Image J (Fiji, https://imagej.nih.gov/ij/). Antibody information is provided in Supp. Data 1.

### Flow cytometry

Mice were anesthetized by CO_2_ asphyxiation and perfused through the circulatory system by injection of 60 ml cold DPBS (Gibco,14190-144). The mouse was quickly decapitated after perfusion and fresh brain tissue was dissected. The brain was dissociated into single-cell suspensions with the Adult Brain Dissociation kit (Miltenyi Biotec,130-107-677). Two million brain cells were seeded per well in a round bottom 96-well plate. Cells were stained with the following panel: the fixable viability dye FVD780, anti-CD45 BUV395, anti-CD11b BV510, anti-Gr1 Pacific Blue, anti-Ly6G BV650, F4/80 BUV661, and MHC II FITC. Flow cytometry was performed with the Cytoflex-LX Flow Cytometer (Beckman Coulter). Data were analyzed using CytExpert software. Antibody information is provided in Supp. Data 1.

### Magnetic resonance imaging (MRI) of young and old mouse brains

#### Data acquisition

Mice were anesthetized by isoflurane inhalation and perfused through the circulatory system by injection of 50 ml cold PBS (Thermo Fisher) followed by 50 ml paraformaldehyde (PFA, Thermo Fisher). The dissected perfused brains were stored overnight in PFA, washed with PBS, and then stored in PBS for ~10 days at 4 °C. Prior to MRI scanning, each specimen was placed in a 10 mm tube, and immersed in perfluoropolyether (Fomblin LC/8, Solvay Solexis, Italy), a proton free fluid void of a proton-MRI signal. Specimens were imaged using a 7T Bruker vertical bore MRI scanner equipped with a microimaging probe and a 10 mm RF coil. For dMRI, 261 image volumes were acquired for each specimen using a 3D echo-planar imaging pulse sequence with the following parameters: echo time = 42 ms; repetition time = 800 ms; number of segments = 10; and isotropic voxel dimension = 75 μm. Data were acquired using a multi-shell acquisition with six directions and three repetitions for b = 200, 500, and 1000 s/mm^2^, 32 directions and 1 repetition for b = 1700 and 3800 s/mm^2^, 56 directions and 1 repetition for b = 6700 s/mm^2^, and 87 directions and 1 repetition for b = 10,000 s/mm^2^. An additional b = 0 s/mm^2^ image was also acquired with reversed phase-encoding for distortion correction purposes. All dMRI data were acquired using δ = 3 ms and Δ = 20 ms. A T_2_-weighted structural image was also acquired using a multi-slice multi-echo sequence with the following parameters: 16 echo times linearly sampled between 10.6 and 169 ms; repetition time = 2000 ms; and the same spatial parameters described for dMRI.

#### Data processing

Diffusion MRI preprocessing was performed using the TORTOISEV4 pipeline (https://github.com/eurotomania/TORTOISEV4). The dMRI data was first denoised using the MP-PCA denoising technique^73^ and subsequently corrected for eddy-currents distortions. During this process, each diffusion-weighted image was initially quadratically^74^ registered to an average b = 0 s/mm^2^ to image, and subsequently registered to a synthetic image generated using the same bval/bvec with either the DTI model (for b-values up to 6000 s/mm^2^) or the MAP-MRI model (for b-values larger than 6000 s/mm^2^). Center frequency drifts were then estimated using a linear regression model^75^ and applied to all data. The final step in processing was susceptibility distortion correction with the DRBUDDI technique^76^, which used both the blip-up and blip-down dMRI data along with the corresponding T2W image for correction. The final dMRI data was generated by concatenating the blip-up and -down datasets.

### Microglia isolation

Fresh brain tissue was quickly dissected after anaesthetization. The brain was dissociated into single-cell suspension with the Adult Brain Dissociation kit (Miltenyi Biotec, 130-107-677). Primary microglia were isolated from the dissociated cells using CD11b (Microglia) MicroBeads (Miltenyi Biotec, 130-093-634) following the manufacturer’s protocol. CD11b+ cells were collected and counted using a Cellometer K2 image cytometer (Nexcelom). Microglia from *n* = 2 mice per age/sex group were pooled into one biological replicate.

### CUT&RUN

CUT&RUN was performed using the CUT&RUN Assay Kit (Cell Signaling Technology, 86652) following the manufacturer’s instructions. About 500,000 microglia from one biological replicate (as defined above) were used for each reaction. The cells and concanavalin A-coated magnetic beads slurry were incubated with 1.5 µg anti-C/EBPB antibody (Santa Cruz, sc-7962) or 0.1 µg anti-YY1 antibody (Cell Signaling Technology, 46395), or 0.1 µg IgG isotype control (Abcam, ab171870) overnight at 4 °C. The pAG-MNase digestion was stopped with a 1X stop buffer. DNA was purified from enriched chromatin samples using the NEB Monarch DNA Cleanup Kit (NEB, T1130) following the manufacturer’s instructions. Libraries were constructed with the NEBNext Ultra™ II DNA Library Prep Kit (NEB, E7645S). The version 1 protocol (https://www.protocols.io/view/library-prep-for-cut-amp-run-with-nebnext-ultra-ii-kxygxm7pkl8j/v1) was used to enrich for sub-nucleosomal fragments. Notable modifications include incubation at 50 °C for 1 h in the end prep step and 65 °C for 10 s in the Annealing/Extension step in PCR cycles. The libraries were size-selected and purified with SPRIselect (Beckman Coulter, B23318), and quality checked on Agilent Bioanalyzer High Sensitivity DNA chip (Agilent Technologies, 5067-4626). The pooled library was sequenced in two rounds, first using a NextSeq 2000 P2 100 cycle kit (mixed with other samples) and then using a NextSeq 2000 P4 XLEAP-SBS 100 cycle kit for a total of 3.6 billion reads.

### Bioinformatic analysis

#### ST data analysis using Partek Flow

After next generation sequencing, we processed FASTQ files and preliminarily assessed quality control metrics on Space Ranger (v2.0.0 for cohort 1 and v2.1.1 for cohort 2, 10X Genomics). The list of QC metrics for each sample is available in the metrics_summary output of Space Ranger and includes mapping, sequencing, and spot quality checks. Additionally, one can view UMI count distribution on tissue section, preliminary clustering and t-SNE projections. All our samples passedthe initial QC check on Space Ranger. The Space Ranger filtered feature-barcode matrix in HDF5 format, and the spatial information (available on GEO, see Data availability section) was then transferred to Partek Flow (v10.0.23.0531 for cohort 1 and 11.0.23.1204 for cohort 2, Partek Inc.)^13^ to perform further quality assessments. The filtered feature-barcode matrix contains only tissue-associated barcodes in MEX format. Rows are features, barcodes are columns, and each element is the number of UMIs associated with a given feature and barcode. This file is typically passed into third-party packages (in our case, Partek) for further filtering, outlier removal, normalization, etc. In Partek, for cohort 1, the barcoded spots were filtered by counts at low.cutoff = 1 and high.cutoff = 38,855, detected genes at low.cutoff = 1 and high.cutoff = 8012, % mitochondrial counts at low.cutoff = 0, high.cutoff = 35%, % ribosomal counts at low.cutoff = 0, high.cutoff = 14%. For cohort 2, the barcoded spots were filtered by counts at low.cutoff = 1 and high.cutoff = 31,732, detected genes at low.cutoff = 1 and high.cutoff = 7515, % mitochondrial counts at low.cutoff = 0, high.cutoff = 48%, % ribosomal counts at low.cutoff = 0, high.cutoff = 18% (representative cohort 1 in Supp. Fig. 1C, D). Filtered mRNAs with a value ≤1.0 in at least 99 % of the cells were excluded for both cohorts. The data after these initial filtering steps contained 82,672 barcoded spots for cohort 1 and 74,899 for cohort 2, with data for 10,109 mRNAs for cohort 1 and 9317 mRNAs for cohort 2 (Supp. Data 3). Next, the data were variance stabilized by log normalization and scaled to counts per million (CPM, add:1.0, log:2.0). PCA was performed, and the top 100 principal components (PCs) were stored. The data were then clustered using graph-based cluster analysis with a clustering resolution set to 0.5. Spots on visium image were classified based on histology and Uniform Manifold Approximation and Projection (UMAP) and annotated to the graph-based clusters. The annotated spots were named based on specific enriched marker mRNAs defined by Allen Mouse Brain Atlas^14^ and assigned to brain regions. Ultimately, we included 67998 barcoded spots for cohort 1 and 57,893 for cohort 2, representing seven brain region clusters.

To determine if our data had batch effects, we merged the samples in cohort 1 (*n* = 2 per age per sex) or cohort 2 (*n* = 5 per age per sex)and performed dimension reduction, clustering, and UMAP construction. We observed that the data was segregated by brain region and not by sample. Further application of batch correction (either scvi, generalized linear model, or Seurat integration) had little effect. We thus concluded that a batch correction was not required for our dataset. Important steps undertaken to prevent batch effects were as follows: (1) we randomized young, middle-aged, and old samples across slides, (2) we used the same permeabilization time and PCR cycles for both cDNA and library amplification across slides, and (3) we pooled all samples (in each of the two cohorts) and sequenced together to minimize platform differences. Furthermore, we used the a priori knowledge of brain anatomy from the Allen Brain Atlas to annotate our histological images and further refine the clusters.

For differential analysis, after initial preprocessing, quality control, CPM normalization, and determination of spatial identities from clusters, we split the normalized counts into seven regions based on their brain region classification. This was done one by one for each sample. At the end of the region classification process, spots were assigned to a defined brain region across all samples. For each region, differentially abundant mRNAs (DARs) were identified based on age groups (young, middle, and old). ANOVA statistics were applied to each age group comparison with sex included as a covariate. Significant DARs affected by age were filtered by FDR using the Benjamini–Hochberg method <0.05, and FC ≥1.5 or ≤−1.5. Visualization of DARs by strip chart and heatmaps was performed in R (v4.3.0) or GraphPad Prism (v9.4.1).

For pseudobulk analysis, we combined spots of all 7 regions from the initial counts post-QC, to represent counts from the entire coronal section and performed CPM normalization. DARs were identified based on age groups (young, middle, and old). ANOVA statistics were applied to each age group comparison for differential analysis with sex included as a covariate. Significant DARs affected by age were filtered by FDR using the Benjamini–Hochberg method <0.05, and FC ≥1.5 or ≤−1.5. Visualization of DARs by volcano plots and heatmaps was performed in R (v4.3.0).

For sex comparisons, we split the CPM normalized counts into female and male groups for each region or pseudobulk. In each sex group, ANOVA statistics were applied to old versus young comparison for differential analysis. Significant DARs affected by age were filtered by FDR using the Benjamini–Hochberg method <0.05, and FC ≥1.5 or ≤−1.5. Visualization of the DARs by age and sex via scatterplots was performed in R (v4.3.0) or GraphPad Prism (v9.4.1).

#### Cell-type identification by RCTD

Cell-type identification was performed using robust cell-type decomposition (RCTD)^26^, a method that learns from single-cell RNA-seq references and decomposes cell-type mixtures in spatial data. RCTD first constructs a single-cell reference (reference) from counts, cell-type information, and nUMIs using the Reference constructor function. Next, it takes the spatial transcriptomics data and loads it into a SpatialRNA object (puck). The RCTD run using the reference and puck generates results stored in the @results field. @results$weights is a data frame of cell-type weights for each spot and can be interpreted as the proportion of RNA molecules originating from each cell type in each spot. Finally, RCTD performs a platform effect normalization step, which normalizes the scRNA-seq cell type profiles to match the platform effects of the spatial transcriptomics dataset.

The reference single-cell RNA-seq data was from ref. ^27^. RCTD (v2.2.1) was run in R (v4.3.0) using the open-source R package at https://github.com/dmcable/RCTD. The doublet_mode argument was set to run RCTD in full mode. The plot_puck_continuous function was used to plot the continuous value over locations on the puck (Supp. Fig. 6A). RCTD outputs a normalized weight for each of the 25 cell types per sample. Normalized weight mean values were calculated by taking the average weight of all spots for each cell type. These mean values were further averaged across two technical replicates for each mouse (*n* = 4 per age group, Supp. Data 8) to determine the cell composition per sample and plotted in Supp. Fig. 6B.

#### Identification of differentially abundant mRNAs by SpatialDE

SpatialDE package (v1.1.0)^15^ in Python (3.8) was used to validate the expression of representative well-known region-specific markers in the brain. The open-source implementation of SpatialDE is available at GitHub (https://github.com/Teichlab/SpatialDE).

#### Analysis of WAM/DAM signatures using SPATA

SPAtial Transcriptomic Analysis (SPATA2)^77^ was performed to visualize WAM and DAM signatures within a spatial context. The SPATA2 (v0.1.0) was run in R (v4.3.0). The source code of SPATA2 (v0.1.0) is available on GitHub (https://github.com/theMILOlab/SPATA).

#### Machine learning

To define input features for the machine learning models, we used ChIP-seq data previously described in ref. ^33^. The ChIP-seq data were converted to a binary representation indicating whether a TF had any binding sites on a gene or not. In total 452 TFs and 5191 mRNAs were used. Differential expression measurements were also codified independently for each brain region based on their FC as up, down, or constant. Differential mRNA results were categorized as up if the FC >1.2 and down if <−1.2. For cohort 1 an FDR <0.05 was used whereas for cohort 2 FDR <0.1 was used. Any mRNA not passing the FDR or FC filter was categorized as constant. A machine learning model was trained to predict the FC class in each region independently. The Random Forest model was used from the R library caret and code previously described in ref. ^33^. Two-thirds of the genes were used as the training partition, while the remaining third was used to test prediction accuracy. 100 models were trained for each region using random initialization, where each model had genes sampled within the training set to ensure equal numbers of the three FC classes. The final prediction was defined as up or down if at least 75% of the models agreed as previously described^33^. Feature importance was estimated by quantifying the reduction in model accuracy when the feature was shuffled in the input.

#### CUT&RUN analysis

CUT&RUN analysis was performed as outlined in Zheng et al., following the protocols.io tutorial (10.17504/protocols.io.bjk2kkye). Briefly, sequencing reads (~107 million paired-end reads per sample) were de-multiplexed to generate compressed FASTQ files by bcl-convert (v4.1.50). Paired-end reads were trimmed using Trim Galore (v0.6.7) to remove the adapter. The qualities of the FASTQs were assessed using FASTQC (v0.11.9). Reads were then aligned to the mouse genome (mm10) using Bowtie2 (v2.5.3) with the parameters -very-sensitive-local, -I 10, and -X 700. SAM output files from Bowtie2 were then filtered to retain alignments with a minimum mapping quality of 2 using samtools (v1.19)^78^. Aligned reads mapping to Encyclopedia of DNA Elements (ENCODE) blacklist regions^79^ were removed using intersect function in bedtools (v2.30.0). RPKM (reads per kilobase per million mapped reads) normalized bigWig files were generated using the bamCoverage function in deepTools (v3.5.4)^80^. Genome browser tracks were created by uploading bigWig files on the University of California, Santa Cruz Genome Browser using custom tracks.

#### Violin plots

Violin plot showing the distribution of UMI counts, detected mRNAs, % mitochondrial counts, and % ribosomal counts of each barcoded spot in each age group was made in Partek Flow (v10.0.23.0531, Partek Inc.)^13^.

#### Volcano plots

Volcano plots were made by ggplot2 package in R (v4.3.0) to show -log_10_(FDR) and log_2_(FC) values for all 10109 mRNAs.

#### Metaplots

Metaplots of CUT&RUN data were generated with deepTools (v3.5.5)^80^. The computeMatrix function was first used to calculate the signal intensity on the bodies of the 73 upregulated genes shared across at least five brain regions (Fig. 2D) and then metaplots drawn with the plotProfile function. Control regions were bodies of 91 downregulated genes (Fig. 2D). bwtool (v1.0)^81^ was used to get genome coverage information (AUC) across the regions of interest and statistical differences were assessed by GraphPad Prism (v10.2.1).

#### Heatmaps

Heatmaps of shared and unique age-related mRNAs were made by Heatmapper (www.heatmapper.ca/expression)^82^. All other heatmaps were made by the pheatmap package in R (v4.3.0).

#### Gene Ontology (GO) analysis

Age-related pathways were identified by gene set enrichment analysis (GSEA)^83^. GSEA (v4.3.2) was used following software documentation. The input was all 10,109 mRNAs which were ranked based on their DAR changes and significance. GO biological processes database was applied as a reference. 1000 random permutations were performed to calculate the *p* values for each pathway. The top ten significant (FDR <0.05) age-related pathways of each comparison were shown in heatmap and bubble plots by normalized enrichment scores (NES).

### Reporting summary

Further information on research design is available in the Nature Portfolio Reporting Summary linked to this article.

## Supplementary information

Supplementary Information

Description of Additional Supplementary Information

Supplementary Data 1

Supplementary Data 2

Supplementary Data 3

Supplementary Data 4

Supplementary Data 5

Supplementary Data 6

Supplementary Data 7

Supplementary Data 8

Supplementary Data 9

Supplementary Data 10

Reporting Summary

Peer Review file

## Data Availability

Raw and processed spatial transcriptomics data and CUT&RUN data generated in this study are available through the NCBI Gene Expression Omnibus (GEO) repository under accession number GSE284202. Source data are provided with this paper and also available at MendeleyData (10.17632/96wzkvjm6n.1).

## Code availability

The code and pipeline for the analyses performed in this study are available at https://github.com/PSenlab/Wang_2025 and published at 10.5281/zenodo.14750569.

## References

[1] Collaborators, G. B. D. D. F. Estimation of the global prevalence of dementia in 2019 and forecasted prevalence in 2050: an analysis for the Global Burden of Disease Study 2019. *Lancet Public Health* **7**, e105–e125 (2022). 34998485 10.1016/S2468-2667(21)00249-8 PMC8810394

[2] Hou, Y. et al. Ageing as a risk factor for neurodegenerative disease. *Nat. Rev. Neurol.* **15**, 565–581 (2019). 31501588 10.1038/s41582-019-0244-7

[3] Bethlehem, R. A. I. et al. Brain charts for the human lifespan. *Nature* **604**, 525–533 (2022). 35388223 10.1038/s41586-022-04554-y PMC9021021

[4] Hall, D. M. et al. Aging reduces adaptive capacity and stress protein expression in the liver after heat stress. *J. Appl Physiol.* **89**, 749–759 (2000). 10926662 10.1152/jappl.2000.89.2.749

[5] Kurosaki, S. et al. Cell fate analysis of zone 3 hepatocytes in liver injury and tumorigenesis. *JHEP Rep.* **3**, 100315 (2021). 34345813 10.1016/j.jhepr.2021.100315 PMC8319533

[6] Lake, B. B. et al. An atlas of healthy and injured cell states and niches in the human kidney. *Nature* **619**, 585–594 (2023). 37468583 10.1038/s41586-023-05769-3 PMC10356613

[7] Wang, Y. X. et al. A single cell spatial temporal atlas of skeletal muscle reveals cellular neighborhoods that orchestrate regeneration and become disrupted in aging. Preprint at *bioRxiv*10.1101/2022.06.10.494732 (2022).

[8] Chen, W. T. et al. Spatial transcriptomics and in situ sequencing to study Alzheimer’s disease. *Cell* **182**, 976–991.e19 (2020). 32702314 10.1016/j.cell.2020.06.038

[9] Lucin, K. M. & Wyss-Coray, T. Immune activation in brain aging and neurodegeneration: too much or too little? *Neuron* **64**, 110–122 (2009). 19840553 10.1016/j.neuron.2009.08.039 PMC2834890

[10] Knox, E. G., Aburto, M. R., Clarke, G., Cryan, J. F. & O’Driscoll, C. M. The blood-brain barrier in aging and neurodegeneration. *Mol. Psychiatry* **27**, 2659–2673 (2022). 35361905 10.1038/s41380-022-01511-z PMC9156404

[11] Rao, A., Barkley, D., Franca, G. S. & Yanai, I. Exploring tissue architecture using spatial transcriptomics. *Nature* **596**, 211–220 (2021). 34381231 10.1038/s41586-021-03634-9 PMC8475179

[12] Moses, L. & Pachter, L. Museum of spatial transcriptomics. *Nat. Methods* **19**, 534–546 (2022). 35273392 10.1038/s41592-022-01409-2

[13] Partek Inc. (2020). Partek® Flow® (Version 10.0.23.0531 or 11.0.23.1204) [Computer software]. https://www.partek.com/partek-flow/ (2020).

[14] Allen Reference Atlas. Mouse brain [brain atlas]. atlas.brain-map.org.

[15] Svensson, V., Teichmann, S. A. & Stegle, O. SpatialDE: identification of spatially variable genes. *Nat. Methods* **15**, 343–346 (2018). 29553579 10.1038/nmeth.4636 PMC6350895

[16] Emos, M. C., Khan Suheb, M. Z. & Agarwal, S. Neuroanatomy, Internal Capsule. *StatPearls* (2024). 31194338

[17] Reuter-Lorenz, P. A. & Stanczak, L. Differential effects of aging on the functions of the corpus callosum. *Dev. Neuropsychol.* **18**, 113–137 (2000). 11143802 10.1207/S15326942DN1801_7

[18] Safaiyan, S. et al. White matter aging drives microglial diversity. *Neuron* **109**, 1100–1117.e10 (2021). 33606969 10.1016/j.neuron.2021.01.027

[19] Keren-Shaul, H. et al. A unique microglia type associated with restricting development of Alzheimer’s disease. *Cell* **169**, 1276–1290 e17 (2017). 28602351 10.1016/j.cell.2017.05.018

[20] Suryadevara, V. et al. SenNet recommendations for detecting senescent cells in different tissues. *Nat. Rev. Mol. Cell Biol*. **25**, 1001–1023 (2024). 10.1038/s41580-024-00738-8 PMC11578798 38831121

[21] Love, S. Demyelinating diseases. *J. Clin. Pathol.* **59**, 1151–1159 (2006). 17071802 10.1136/jcp.2005.031195 PMC1860500

[22] Peters, A. The effects of normal aging on myelin and nerve fibers: a review. *J. Neurocytol.* **31**, 581–593 (2002). 14501200 10.1023/a:1025731309829

[23] Ozarslan, E. et al. Mean apparent propagator (MAP) MRI: a novel diffusion imaging method for mapping tissue microstructure. *Neuroimage* **78**, 16–32 (2013). 23587694 10.1016/j.neuroimage.2013.04.016 PMC4059870

[24] Bouhrara, M., Avram, A. V., Kiely, M., Trivedi, A. & Benjamini, D. Adult lifespan maturation and degeneration patterns in gray and white matter: a mean apparent propagator (MAP) MRI study. *Neurobiol. Aging* **124**, 104–116 (2023). 36641369 10.1016/j.neurobiolaging.2022.12.016 PMC9985137

[25] Spotorno, N. et al. Measures of cortical microstructure are linked to amyloid pathology in Alzheimer’s disease. *Brain* **146**, 1602–1614 (2023). 36130332 10.1093/brain/awac343 PMC10115177

[26] Cable, D. M. et al. Robust decomposition of cell type mixtures in spatial transcriptomics. *Nat. Biotechnol.* **40**, 517–526 (2022). 33603203 10.1038/s41587-021-00830-w PMC8606190

[27] Ximerakis, M. et al. Single-cell transcriptomic profiling of the aging mouse brain. *Nat. Neurosci.* **22**, 1696–1708 (2019). 31551601 10.1038/s41593-019-0491-3

[28] Cho, R. H., Sieburg, H. B. & Muller-Sieburg, C. E. A new mechanism for the aging of hematopoietic stem cells: aging changes the clonal composition of the stem cell compartment but not individual stem cells. *Blood* **111**, 5553–5561 (2008). 18413859 10.1182/blood-2007-11-123547 PMC2424153

[29] Kim, H. et al. Reactive astrocytes transduce inflammation in a blood-brain barrier model through a TNF-STAT3 signaling axis and secretion of alpha 1-antichymotrypsin. *Nat. Commun.* **13**, 6581 (2022). 36323693 10.1038/s41467-022-34412-4 PMC9630454

[30] Marques, F., Sousa, J. C., Sousa, N. & Palha, J. A. Blood-brain-barriers in aging and in Alzheimer’s disease. *Mol. Neurodegener.* **8**, 38 (2013). 24148264 10.1186/1750-1326-8-38 PMC4015275

[31] Zenaro, E. et al. Neutrophils promote Alzheimer’s disease-like pathology and cognitive decline via LFA-1 integrin. *Nat. Med.* **21**, 880–886 (2015). 26214837 10.1038/nm.3913

[32] Benayoun, B. A. et al. Remodeling of epigenome and transcriptome landscapes with aging in mice reveals widespread induction of inflammatory responses. *Genome Res.* **29**, 697–709 (2019). 30858345 10.1101/gr.240093.118 PMC6442391

[33] Lu, R. J. et al. Multi-omic profiling of primary mouse neutrophils predicts a pattern of sex and age-related functional regulation. *Nat. Aging* **1**, 715–733 (2021). 34514433 10.1038/s43587-021-00086-8 PMC8425468

[34] Qin, Q. et al. Lisa: inferring transcriptional regulators through integrative modeling of public chromatin accessibility and ChIP-seq data. *Genome Biol.* **21**, 32 (2020). 32033573 10.1186/s13059-020-1934-6 PMC7007693

[35] Lu, Y. et al. Regulation of TREM2 expression by transcription factor YY1 and its protective effect against Alzheimer’s disease. *J. Biol. Chem.* **299**, 104688 (2023). 37044212 10.1016/j.jbc.2023.104688 PMC10193014

[36] Troutman, T. D., Kofman, E. & Glass, C. K. Exploiting dynamic enhancer landscapes to decode macrophage and microglia phenotypes in health and disease. *Mol. Cell* **81**, 3888–3903 (2021). 34464593 10.1016/j.molcel.2021.08.004 PMC8500948

[37] Skene, P. J. & Henikoff, S. An efficient targeted nuclease strategy for high-resolution mapping of DNA binding sites. *Elife* **6**, e21856 (2017). 10.7554/eLife.21856 PMC5310842 28079019

[38] Bohlen, C. J. et al. Diverse requirements for microglial survival, specification, and function revealed by defined-medium cultures. *Neuron* **94**, 759–773 e8 (2017). 28521131 10.1016/j.neuron.2017.04.043 PMC5523817

[39] Prah, J. et al. A novel serum free primary astrocyte culture method that mimic quiescent astrocyte phenotype. *J. Neurosci. Methods* **320**, 50–63 (2019). 30904500 10.1016/j.jneumeth.2019.03.013 PMC6546087

[40] Hajdarovic, K. H. et al. Single-cell analysis of the aging female mouse hypothalamus. *Nat. Aging* **2**, 662–678 (2022). 36285248 10.1038/s43587-022-00246-4 PMC9592060

[41] Murdock, M. H. & Tsai, L. H. Insights into Alzheimer’s disease from single-cell genomic approaches. *Nat. Neurosci.* **26**, 181–195 (2023). 36593328 10.1038/s41593-022-01222-2 PMC10155598

[42] Suda, M. et al. Senolytic vaccination improves normal and pathological age-related phenotypes and increases lifespan in progeroid mice. *Nat. Aging* **1**, 1117–1126 (2021). 37117524 10.1038/s43587-021-00151-2

[43] Huttenrauch, M. et al. Glycoprotein NMB: a novel Alzheimer’s disease associated marker expressed in a subset of activated microglia. *Acta Neuropathol. Commun.* **6**, 108 (2018). 30340518 10.1186/s40478-018-0612-3 PMC6194687

[44] Ko, C. Y. et al. CCAAT/enhancer binding protein delta (CEBPD) elevating PTX3 expression inhibits macrophage-mediated phagocytosis of dying neuron cells. *Neurobiol. Aging* **33**, 422 e11–422 e25 (2012). 21112127 10.1016/j.neurobiolaging.2010.09.017 PMC6309870

[45] Hahn, O. et al. Atlas of the aging mouse brain reveals white matter as vulnerable foci. *Cell* **186**, 4117–4133 e22 (2023). 37591239 10.1016/j.cell.2023.07.027 PMC10528304

[46] Bouhrara, M. et al. Age-related estimates of aggregate g-ratio of white matter structures assessed using quantitative magnetic resonance neuroimaging. *Hum. Brain Mapp.* **42**, 2362–2373 (2021). 33595168 10.1002/hbm.25372 PMC8090765

[47] Walker, K. A. et al. MRI and fluid biomarkers reveal determinants of myelin and axonal loss with aging. *Ann. Clin. Transl. Neurol.* **10**, 397–407 (2023). 36762407 10.1002/acn3.51730 PMC10014005

[48] Lawrence, K. E. et al. Age and sex effects on advanced white matter microstructure measures in 15,628 older adults: a UK biobank study. *Brain Imaging Behav.* **15**, 2813–2823 (2021). 34537917 10.1007/s11682-021-00548-y PMC8761720

[49] Sullivan, E. V. & Pfefferbaum, A. Diffusion tensor imaging and aging. *Neurosci. Biobehav. Rev.* **30**, 749–761 (2006). 16887187 10.1016/j.neubiorev.2006.06.002

[50] Schilling, K. G. et al. Aging and white matter microstructure and macrostructure: a longitudinal multi-site diffusion MRI study of 1218 participants. *Brain Struct. Funct.* **227**, 2111–2125 (2022). 35604444 10.1007/s00429-022-02503-z PMC9648053

[51] Allen, W. E., Blosser, T. R., Sullivan, Z. A., Dulac, C. & Zhuang, X. Molecular and spatial signatures of mouse brain aging at single-cell resolution. *Cell* **186**, 194–208 e18 (2023). 36580914 10.1016/j.cell.2022.12.010 PMC10024607

[52] Chia, L. S., Thompson, J. E. & Moscarello, M. A. Changes in lipid phase behaviour in human myelin during maturation and aging. Involvement of lipid peroxidation. *FEBS Lett.* **157**, 155–158 (1983). 6862012 10.1016/0014-5793(83)81136-3

[53] Thomas, B. P., Liu, P., Park, D. C., van Osch, M. J. & Lu, H. Cerebrovascular reactivity in the brain white matter: magnitude, temporal characteristics, and age effects. *J. Cereb. Blood Flow. Metab.* **34**, 242–247 (2014). 24192640 10.1038/jcbfm.2013.194 PMC3915204

[54] Juttukonda, M. R. et al. Characterizing cerebral hemodynamics across the adult lifespan with arterial spin labeling MRI data from the Human Connectome Project-Aging. *Neuroimage* **230**, 117807 (2021). 33524575 10.1016/j.neuroimage.2021.117807 PMC8185881

[55] Yu, F. et al. Phagocytic microglia and macrophages in brain injury and repair. *CNS Neurosci. Ther.* **28**, 1279–1293 (2022). 35751629 10.1111/cns.13899 PMC9344092

[56] Diaz-Alvarez, L. & Ortega, E. The many roles of galectin-3, a multifaceted molecule, in innate immune responses against pathogens. *Mediators Inflamm.* **2017**, 9247574 (2017). 28607536 10.1155/2017/9247574 PMC5457773

[57] Perry, V. H. & Holmes, C. Microglial priming in neurodegenerative disease. *Nat. Rev. Neurol.* **10**, 217–224 (2014). 24638131 10.1038/nrneurol.2014.38

[58] Krasemann, S. et al. The TREM2-APOE pathway drives the transcriptional phenotype of dysfunctional microglia in neurodegenerative diseases. *Immunity* **47**, 566–58V (2017). 28930663 10.1016/j.immuni.2017.08.008 PMC5719893

[59] Guerreiro, R. et al. TREM2 variants in Alzheimer’s disease. *N. Engl. J. Med*. **368**, 117–127 (2013). 23150934 10.1056/NEJMoa1211851 PMC3631573

[60] Garcia-Revilla, J. et al. Galectin-3, a rising star in modulating microglia activation under conditions of neurodegeneration. *Cell Death Dis.* **13**, 628 (2022). 35859075 10.1038/s41419-022-05058-3 PMC9300700

[61] Boza-Serrano, A. et al. Galectin-3, a novel endogenous TREM2 ligand, detrimentally regulates inflammatory response in Alzheimer’s disease. *Acta Neuropathol.* **138**, 251–273 (2019). 31006066 10.1007/s00401-019-02013-z PMC6660511

[62] Jiang, H. R. et al. Galectin-3 deficiency reduces the severity of experimental autoimmune encephalomyelitis. *J. Immunol.* **182**, 1167–1173 (2009). 19124760 10.4049/jimmunol.182.2.1167

[63] Burguillos, M. A. et al. Microglia-secreted galectin-3 acts as a Toll-like receptor 4 ligand and contributes to microglial activation. *Cell Rep.* **10**, 1626–1638 (2015). 25753426 10.1016/j.celrep.2015.02.012

[64] MacKinnon, A. C. et al. Regulation of alternative macrophage activation by galectin-3. *J. Immunol.* **180**, 2650–2658 (2008). 18250477 10.4049/jimmunol.180.4.2650

[65] Stephan, A. H. et al. A dramatic increase of C1q protein in the CNS during normal aging. *J. Neurosci.* **33**, 13460–13474 (2013). 23946404 10.1523/JNEUROSCI.1333-13.2013 PMC3742932

[66] Wang, C. et al. Microglia mediate forgetting via complement-dependent synaptic elimination. *Science* **367**, 688–694 (2020). 32029629 10.1126/science.aaz2288

[67] Ingram, G. et al. Complement activation in multiple sclerosis plaques: an immunohistochemical analysis. *Acta Neuropathol. Commun.* **2**, 53 (2014). 24887075 10.1186/2051-5960-2-53 PMC4048455

[68] Loveless, S. et al. Tissue microarray methodology identifies complement pathway activation and dysregulation in progressive multiple sclerosis. *Brain Pathol.* **28**, 507–520 (2018). 28707765 10.1111/bpa.12546 PMC8028318

[69] Morgan, B. P., Gommerman, J. L. & Ramaglia, V. An “outside-in” and “inside-out” consideration of complement in the multiple sclerosis brain: lessons from development and neurodegenerative diseases. *Front. Cell Neurosci.* **14**, 600656 (2020). 33488361 10.3389/fncel.2020.600656 PMC7817777

[70] Bettcher, B. M., Tansey, M. G., Dorothee, G. & Heneka, M. T. Peripheral and central immune system crosstalk in Alzheimer disease - a research prospectus. *Nat. Rev. Neurol.* **17**, 689–701 (2021). 34522039 10.1038/s41582-021-00549-x PMC8439173

[71] Berchtold, N. C. et al. Gene expression changes in the course of normal brain aging are sexually dimorphic. *Proc. Natl Acad. Sci. USA* **105**, 15605–15610 (2008). 18832152 10.1073/pnas.0806883105 PMC2563070

[72] Corrada, M. M., Brookmeyer, R., Berlau, D., Paganini-Hill, A. & Kawas, C. H. Prevalence of dementia after age 90: results from the 90+ study. *Neurology* **71**, 337–343 (2008). 18596243 10.1212/01.wnl.0000310773.65918.cd

[73] Veraart, J., Fieremans, E. & Novikov, D. S. Diffusion MRI noise mapping using random matrix theory. *Magn. Reson. Med.* **76**, 1582–1593 (2016). 26599599 10.1002/mrm.26059 PMC4879661

[74] Rohde, G. K., Barnett, A. S., Basser, P. J., Marenco, S. & Pierpaoli, C. Comprehensive approach for correction of motion and distortion in diffusion-weighted MRI. *Magn. Reson. Med.* **51**, 103–114 (2004). 14705050 10.1002/mrm.10677

[75] Vos, S. B. et al. The importance of correcting for signal drift in diffusion MRI. *Magn. Reson. Med.* **77**, 285–299 (2017). 26822700 10.1002/mrm.26124

[76] Irfanoglu, M. O. et al. DR-BUDDI (diffeomorphic registration for blip-up blip-down diffusion imaging) method for correcting echo planar imaging distortions. *Neuroimage* **106**, 284–299 (2015). 25433212 10.1016/j.neuroimage.2014.11.042 PMC4286283

[77] Ravi, V. M. et al. Spatially resolved multi-omics deciphers bidirectional tumor-host interdependence in glioblastoma. *Cancer Cell* **40**, 639–655 e13 (2022). 35700707 10.1016/j.ccell.2022.05.009

[78] Li, H. et al. The sequence alignment/map format and SAMtools. *Bioinformatics* **25**, 2078–2079 (2009). 19505943 10.1093/bioinformatics/btp352 PMC2723002

[79] Amemiya, H. M., Kundaje, A. & Boyle, A. P. The ENCODE blacklist: identification of problematic regions of the genome. *Sci. Rep.* **9**, 9354 (2019). 31249361 10.1038/s41598-019-45839-z PMC6597582

[80] Ramirez, F., Dundar, F., Diehl, S., Gruning, B. A. & Manke, T. deepTools: a flexible platform for exploring deep-sequencing data. *Nucleic Acids Res.* **42**, W187–W191 (2014). 24799436 10.1093/nar/gku365 PMC4086134

[81] Pohl, A. & Beato, M. bwtool: a tool for bigWig files. *Bioinformatics* **30**, 1618–1619 (2014). 24489365 10.1093/bioinformatics/btu056 PMC4029031

[82] Babicki, S. et al. Heatmapper: web-enabled heat mapping for all. *Nucleic Acids Res.* **44**, W147–W153 (2016). 27190236 10.1093/nar/gkw419 PMC4987948

[83] Subramanian, A. et al. Gene set enrichment analysis: a knowledge-based approach for interpreting genome-wide expression profiles. *Proc. Natl Acad. Sci. USA* **102**, 15545–15550 (2005). 16199517 10.1073/pnas.0506580102 PMC1239896

