# Supplementary material for: Spatial transcriptomics of the aging mouse brain reveals origins of inflammation in the white matter
Source: Nat Commun. 2025 Apr 4;16:3231. doi: 10.1038/s41467-025-58466-2 (PMC11971433; doi:10.1038/s41467-025-58466-2)
Supplement: Supplementary file 1 — Supplementary Information [file 41467_2025_58466_MOESM1_ESM.pdf]

Spatial transcriptomics of the aging mouse brain reveals origins of inflammation in the white matter

Lin Wang[1], Chang-Yi Cui[1], Christopher T. Lee[1], Monica Bodogai[2], Na Yang[1], Changyou Shi[1], Mustafa O. Irfanoglu[3], James R. Occean[1*], Sadia Afrin[1], Nishat Sarker[1], Ross A. McDevitt[4], Elin Lehrmann[5], Shahroze Abbas[6], Nirad Banskota[5], Jinshui Fan[5], Supriyo De[5], Peter Rapp[7], Arya Biragyn[2], Dan Benjamini[7], Manolis Maragkakis[1], Payel Sen[1**].

[1]Laboratory of Genetics and Genomics, National Institute on Aging, NIH, Baltimore, MD, USA

[2]Laboratory of Molecular Biology and Immunology, National Institute on Aging, NIH, Baltimore, MD, USA

[3]Quantitative Medical Imaging Section, National Institute of Biomedical Imaging and Bioengineering, NIH, Bethesda, MD, USA

[4]Comparative Medicine Section, National Institute on Aging, NIH, Baltimore, MD, USA

[5]Computational Biology and Genomics Core, Laboratory of Genetics and Genomics, National Institute on Aging, NIH, Baltimore, MD, USA

[6]Center for Alzheimer's and Related Dementia, National Institute on Aging, NIH, Bethesda, MD, USA

[7]Laboratory of Behavioral Neuroscience, National Institute on Aging, NIH, Baltimore, MD, USA

* Present address: Cancer Biology Program, Stanford University School of Medicine, Stanford, CA, USA

** **Correspondence:** payel.sen@nih.gov

Supp. Figure 1

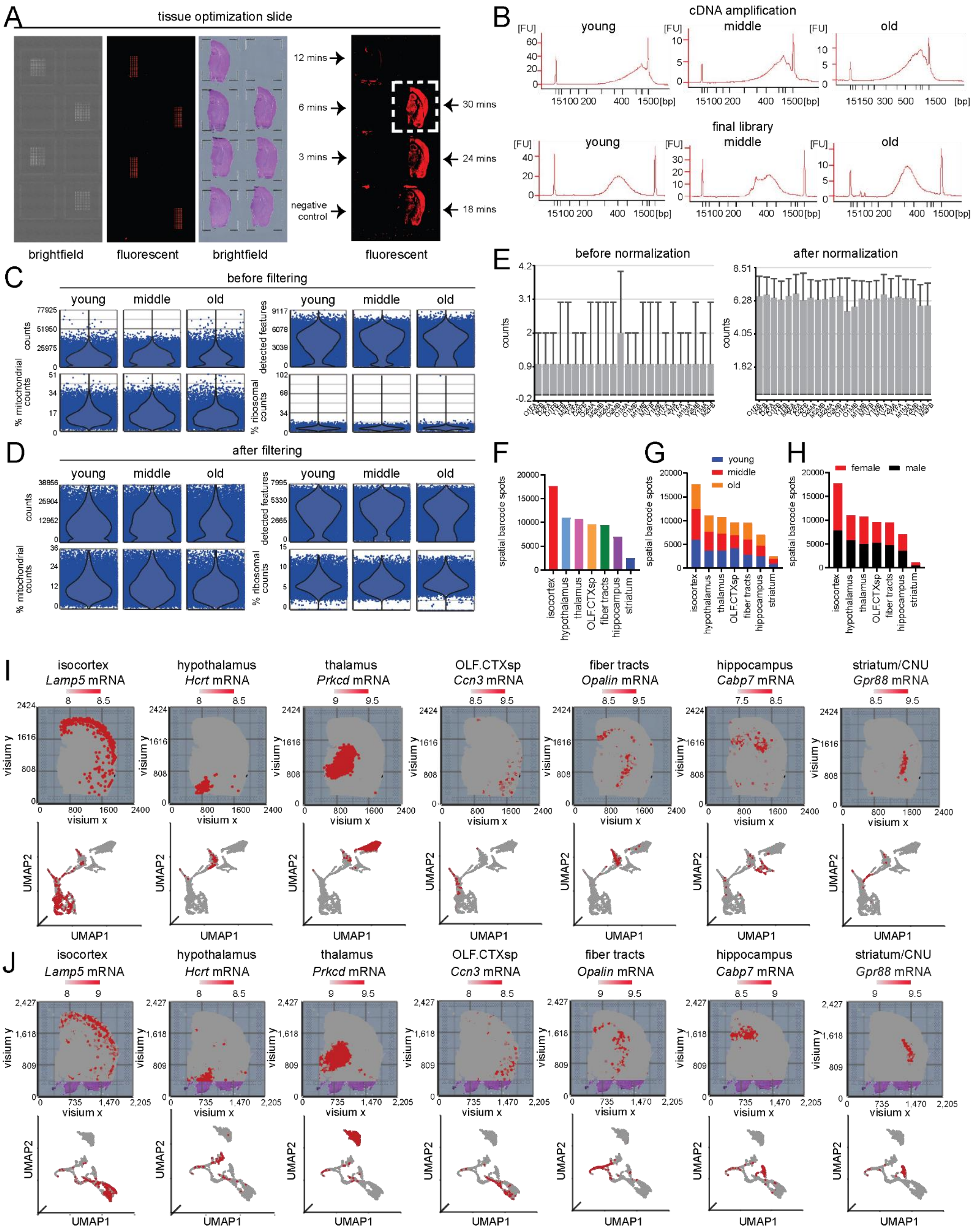

Supp. Figure 1: Permeabilization optimization and QA/QC of dataset (related to Fig. 1).

(A) Brightfield (panel 1) and fluorescence (panel 2) images showing microscope settings on the Visium tissue optimization test slide. Brightfield (panel 3) and fluorescence (panel 4) images after tissue placement and permeabilization for 3, 6, 12, 18, 24 and 30 minutes. Note, optimum signal is obtained at 30 minutes (white dashed box). **(B)** Representative BioAnalyzer traces showing size distribution post-cDNA amplification (top) and final libraries (bottom) of samples in young (Y2FA), middle (M1FB) and old (O2FB). **(C)** Violin plot showing distribution of UMI counts, detected features, % mitochondrial counts, and % ribosomal counts of each barcoded spot in the samples before filtering steps (see Methods). **(D)** Same as (C), except after filtering. **(E)** Box plot showing 10th, 25th, 50th, 75th and 90th percentiles of counts before (top) and after normalization (bottom). A counts per million (CPM) normalization method was used. **(F)** Bar plot showing the total number of spatially barcoded spots of each region across all 24 samples. **(G)** Same as (F) except split on different age groups. **(H)** Same as (F) except split on sex. **(I)** Visium images (top) and UMAP (bottom) visualization of 7 major regions showing the expression of representative well-known region-specific marker mRNAs in a young female sample (Y2FB). Numbers in the color scale reflect the number of UMIs detected for the specified mRNA for each spot. **(J)** Same as (I) except for a middle-aged female sample (M2FA). Source data are provided as a Source Data file.

Supp. Figure 2

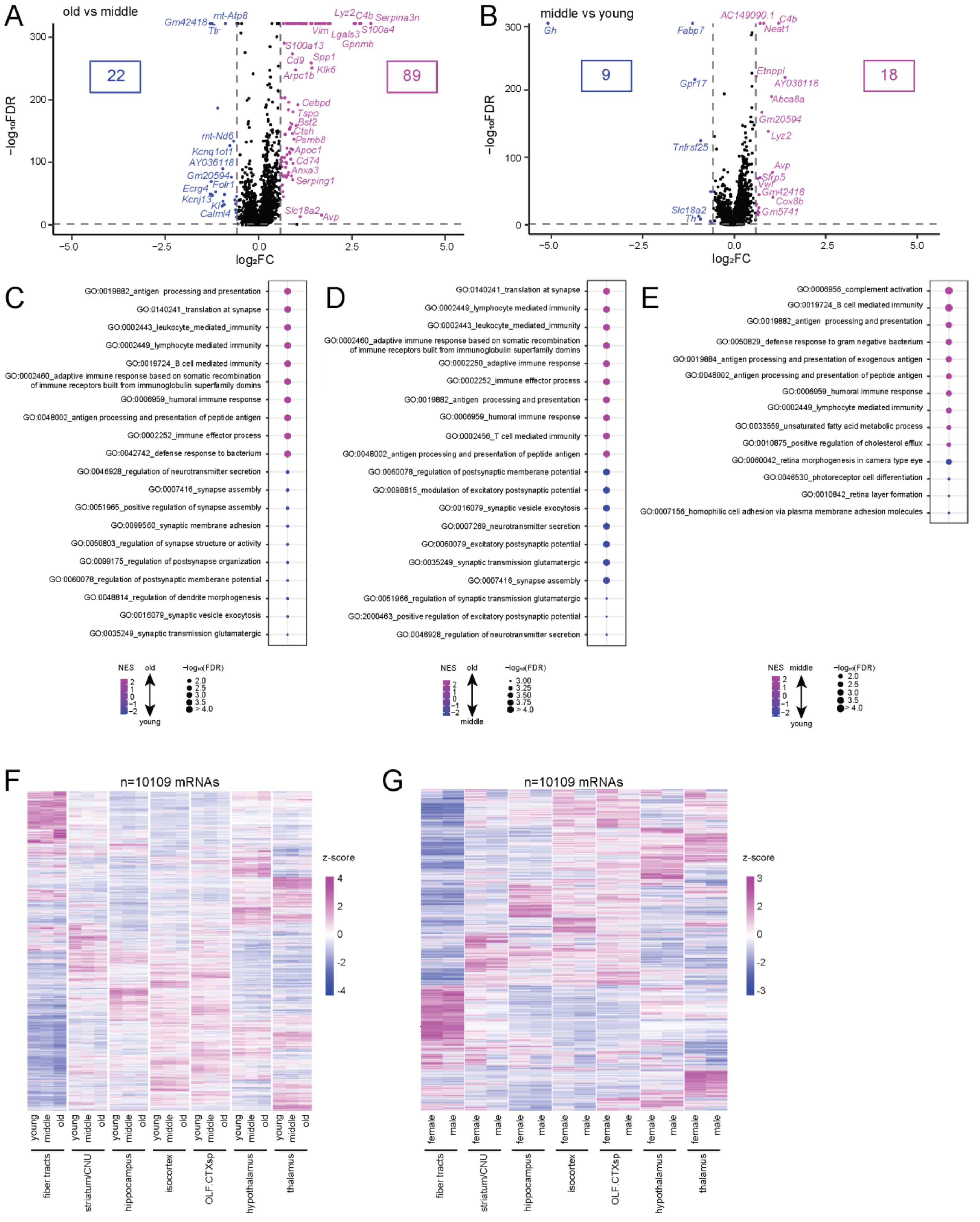

Supp. Figure 2: Anatomical regions of the brain display both shared and unique sets of age-correlated mRNAs (related to Fig. 2).

(A) Volcano plot showing -$\log_{10}$(FDR) and $\log_2$(FC) values for all 10109 mRNAs with 89 significantly upregulated DARs (purple dots; FDR < 0.05, FC ≥ 1.5) and 22 significantly downregulated DARs (blue dots; FDR < 0.05, FC ≤ -1.5) in old vs. middle. **(B)** Volcano plot showing -$\log_{10}$(FDR) and $\log_2$(FC) values for all 10109 mRNAs with 18 significantly upregulated DARs (purple dots; FDR < 0.05, FC ≥ 1.5) and 9 significantly downregulated DARs (blue dots; FDR < 0.05, FC ≤ -1.5) in middle vs. young. For (A-B), some top up- or downregulated mRNAs are labeled. **(C)** Bubble plot showing the top 10 significant (FDR < 0.05) age-related GSEA pathways in old vs. young. **(D)** Same as (C) except in old vs. middle. **(E)** Same as (C) except in middle vs. young. Numbers in the color scale are normalized enrichment scores (NES). Positive NES values indicate upregulated pathways, while negative NES indicate downregulated pathways. The bubble size indicates -$\log_{10}$(FDR) q values. **(F)** Heatmap of count mean values in young, middle-aged, and old samples for all 10109 mRNAs in each region. **(G)** Heatmap of count mean values in male and female samples for all 10109 mRNAs in each region. Source data are provided as a Source Data file.

Supp. Figure 3

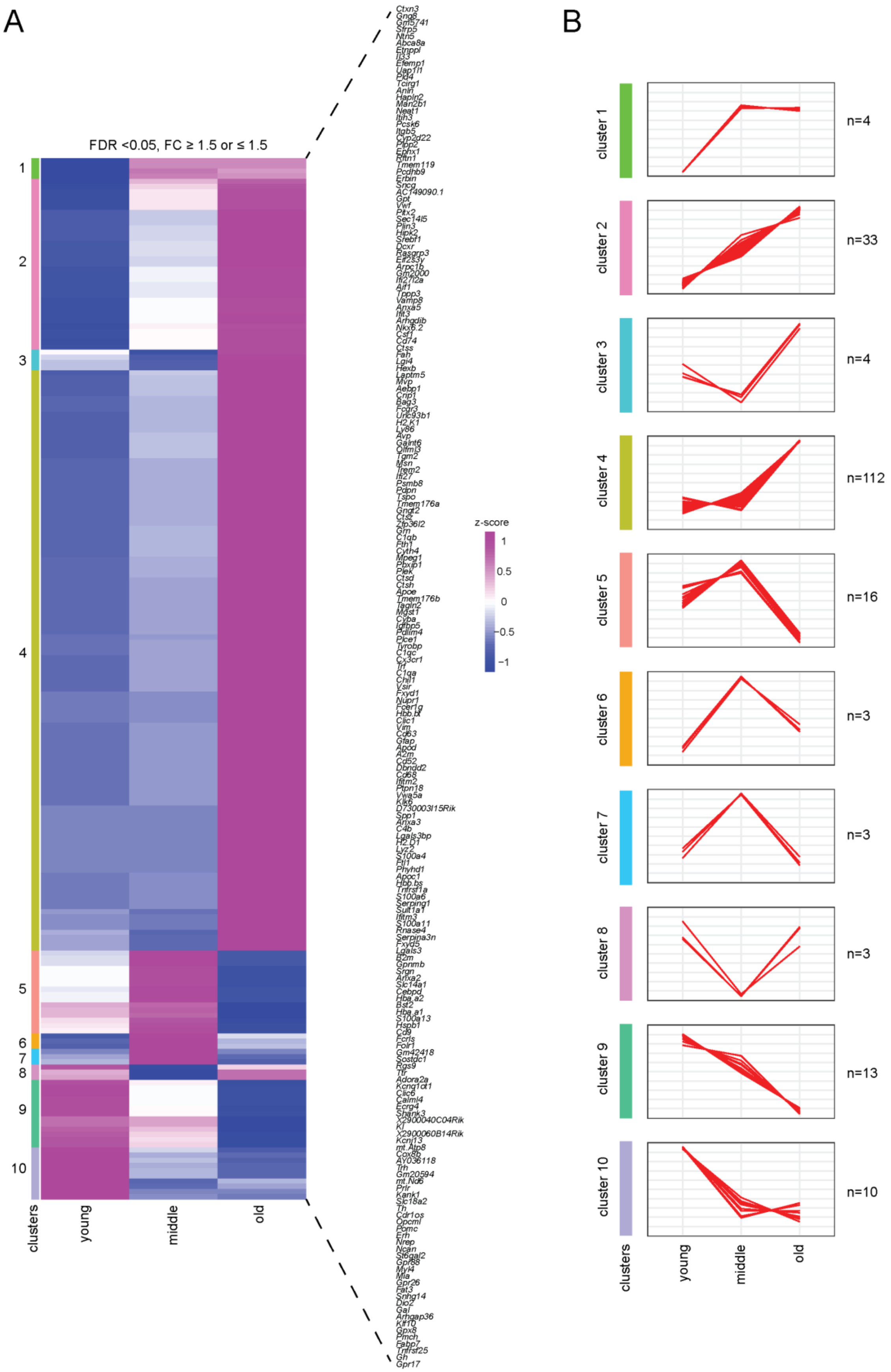

Supp. Figure 3: Inflammatory changes in the aging brain occur late in life (related to Fig. 2).

(A) Clustered heatmap of count mean values in young, middle, and old mice for age-related genes at the pseudobulk level. The age-related mRNAs were filtered by $FDR < 0.05$ and $FC \geq 1.5$ or $FC \leq 1.5$. Clusters are colored on the left. **(B)** Line graphs depicting kinetics of mRNA abundance changes during aging for each cluster. Source data are provided as a Source Data file.

Supp. Figure 4

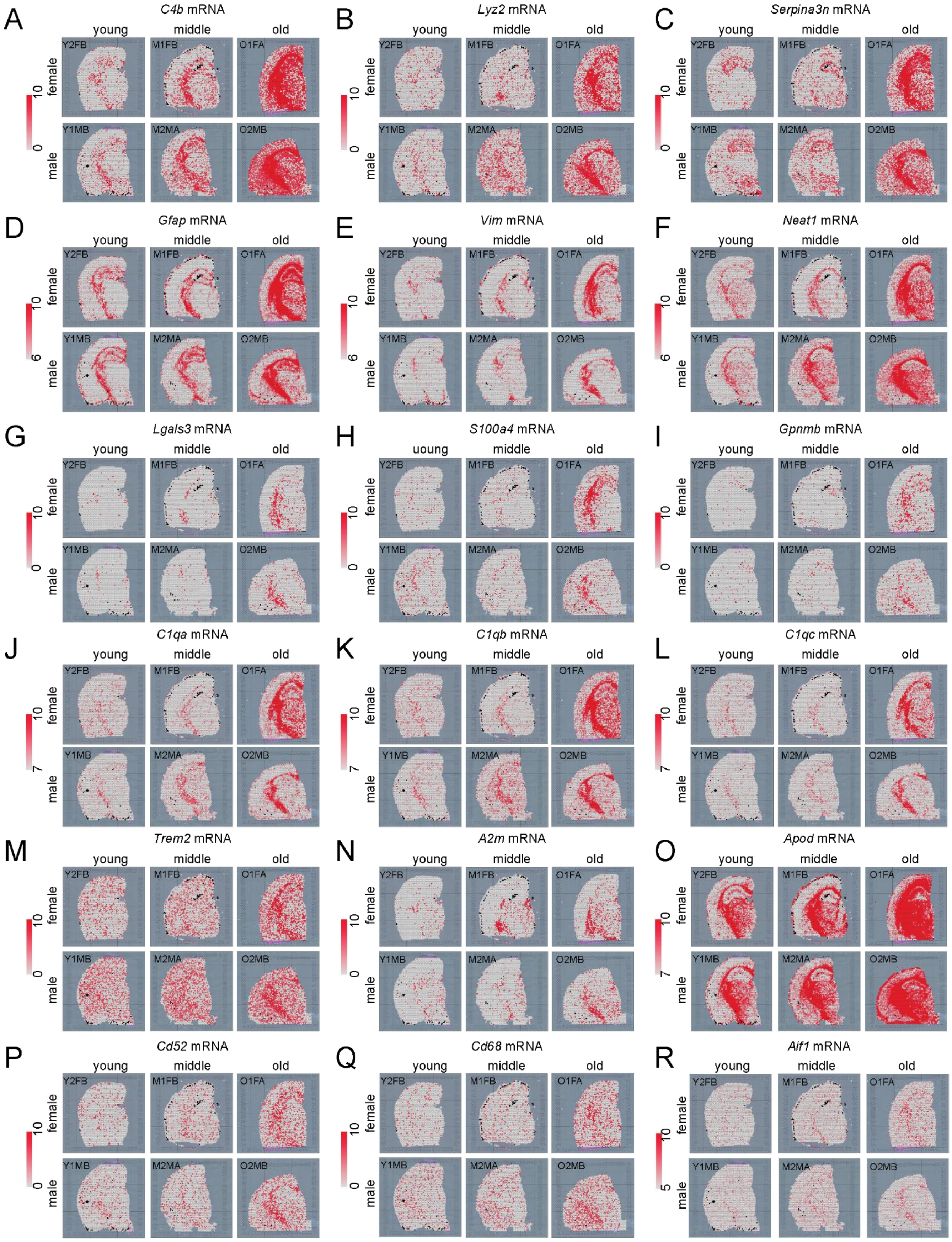

Supp. Figure 4: Annotated Visium images of select shared upregulated DARs (related to Fig. 2).

Visualization of the average expression of select strongly upregulated DARs in young, middle, and old brain sections; **(A)** *C4b*, **(B)** *Lyz2*, **(C)** *Serpina3n*, **(D)** *Gfap*, **(E)** *Vim*, **(F)** *Neat1*, **(G)** *Lgals3*, **(H)** *S100a4*, **(I)** *Gpnmb*, **(J)** *C1qa*, **(K)** *C1qb*, **(L)** *C1qc*, **(M)** *Trem2*, **(N)** *A2m*, **(O)** *Apod*, **(P)** *Cd52*, **(Q)** *Cd68*, and **(R)** *Aif1* mRNA. Numbers in the color scale reflect the number of UMIs detected for the specified mRNA for each spot. Source data are provided as a Source Data file.

Supp. Figure 5

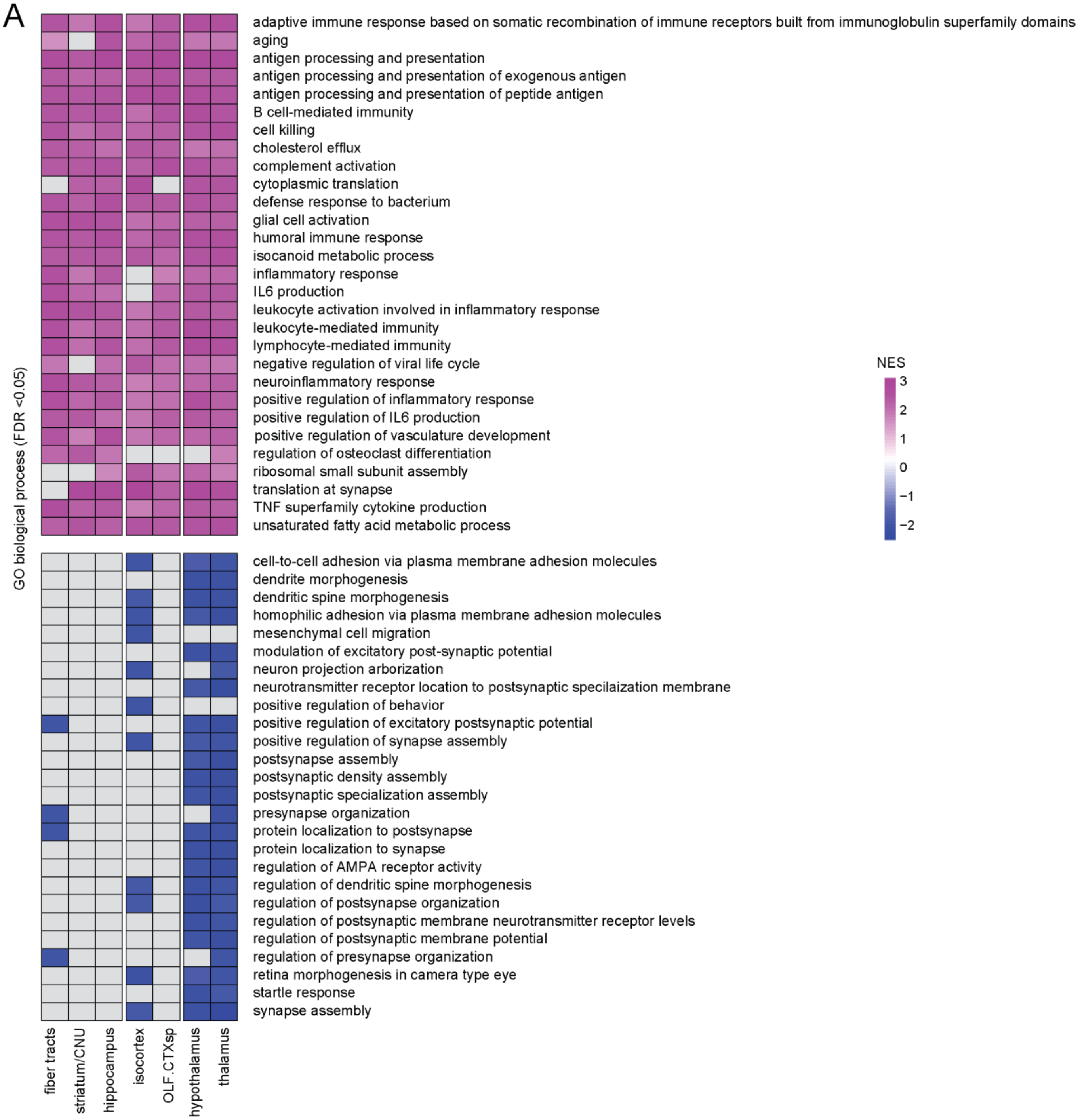

Supp. Figure 5: Aging fiber tracts exhibit signs of immune activation (related to Figs. 2-6).

(A) GSEA of showing the top 10 significant (FDR < 0.05) age-related pathways in each brain region. Combined list is shown in the heatmap, full list and statistics is available in Supp. Table 6. Numbers in the color scale are normalized enrichment scores (NES). Positive NES values indicate upregulated pathways, while negative NES indicate downregulated pathways. Gray cells indicate no significant change with aging. Source data are provided as a Source Data file.

Supp. Figure 6

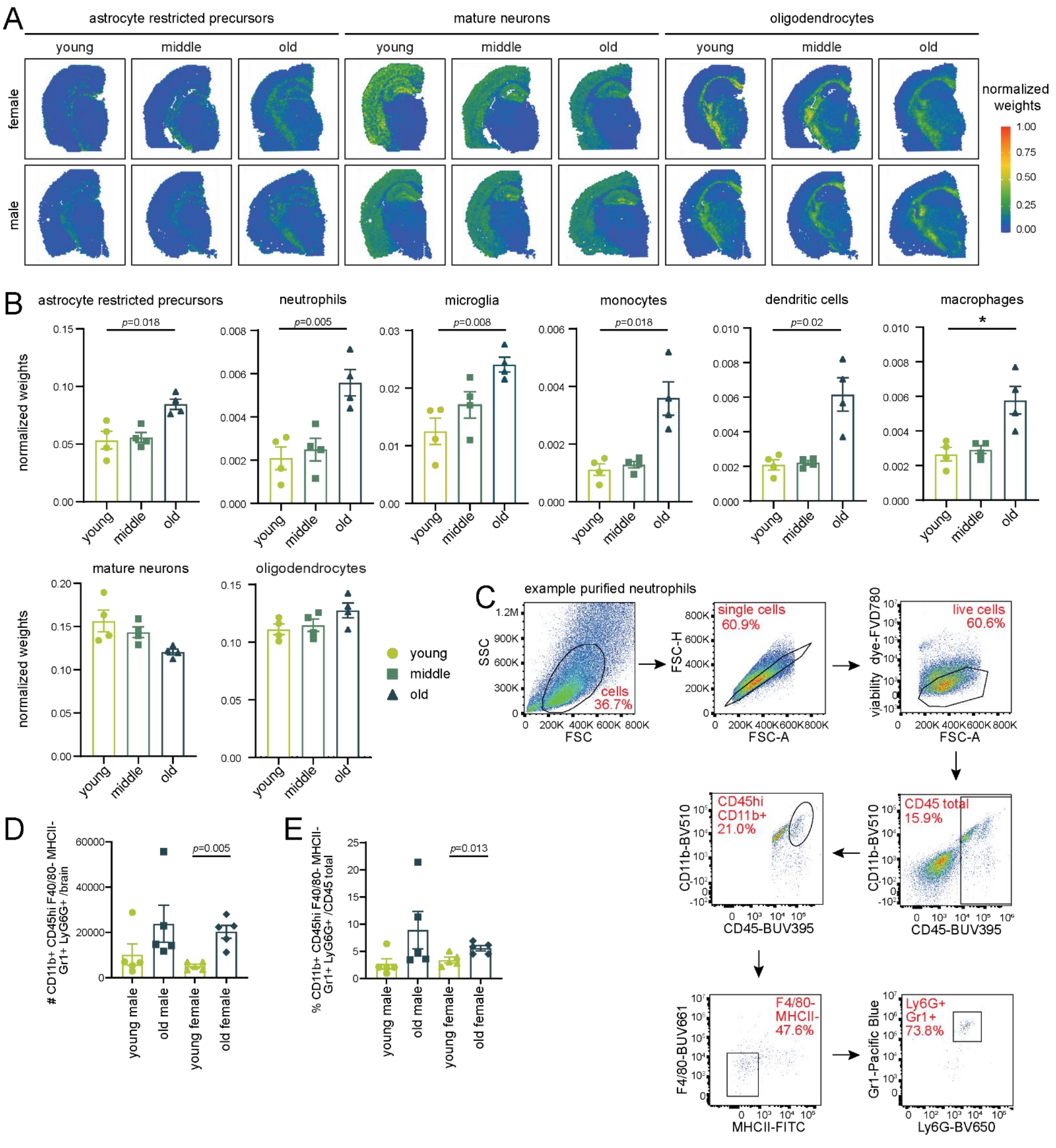

Supp. Figure 6: Innate myeloid immune cells are increased in the old mouse brain (related to Figs. 2-6). **(A)** Plots showing RCTD normalized weights for ARPs (left), mature neurons (middle) and oligodendrocytes (right) in indicated mouse brain samples. Values in the color scale are the normalized weights of cell types from RCTD. **(B)** Bar plot showing RCTD normalized weight mean values of ARPs, neutrophils, microglia, monocytes, dendritic cells, macrophages, mature neurons, and oligodendrocytes across ages. The normalized weight mean values were calculated by taking the average weight of all spots for each cell type. Each dot indicates the average normalized weight mean of two technical replicates for each mouse (n = 4 per age groups). Data are summarized as mean ± standard error of the normalized weight mean values. *p* values are reported from an unpaired two-tailed t-test with Welch's correction of old vs. young and middle vs. young. **(C)** Representative flow cytometry gating strategy for neutrophils purified from a young male mouse (16 weeks). Neutrophils are expected to be double positive for Gr1 and Ly6G. **(D)** Proportion of neutrophils among total CD45-positive cells in young and old, male and female mice (n = 5 for all groups). **(E)** Bar plot showing neutrophil numbers per brain in aged male vs. female mice (n = 5 for all groups), determined as Gr1 and Ly6G double positive population. For (D-E), data are summarized as mean ± standard error of the mean number. *p* values are reported from an unpaired two-tailed t-test with Welch's correction of old female vs. young female and old male vs. young male. Source data are provided as a Source Data file.

Supp. Figure 7

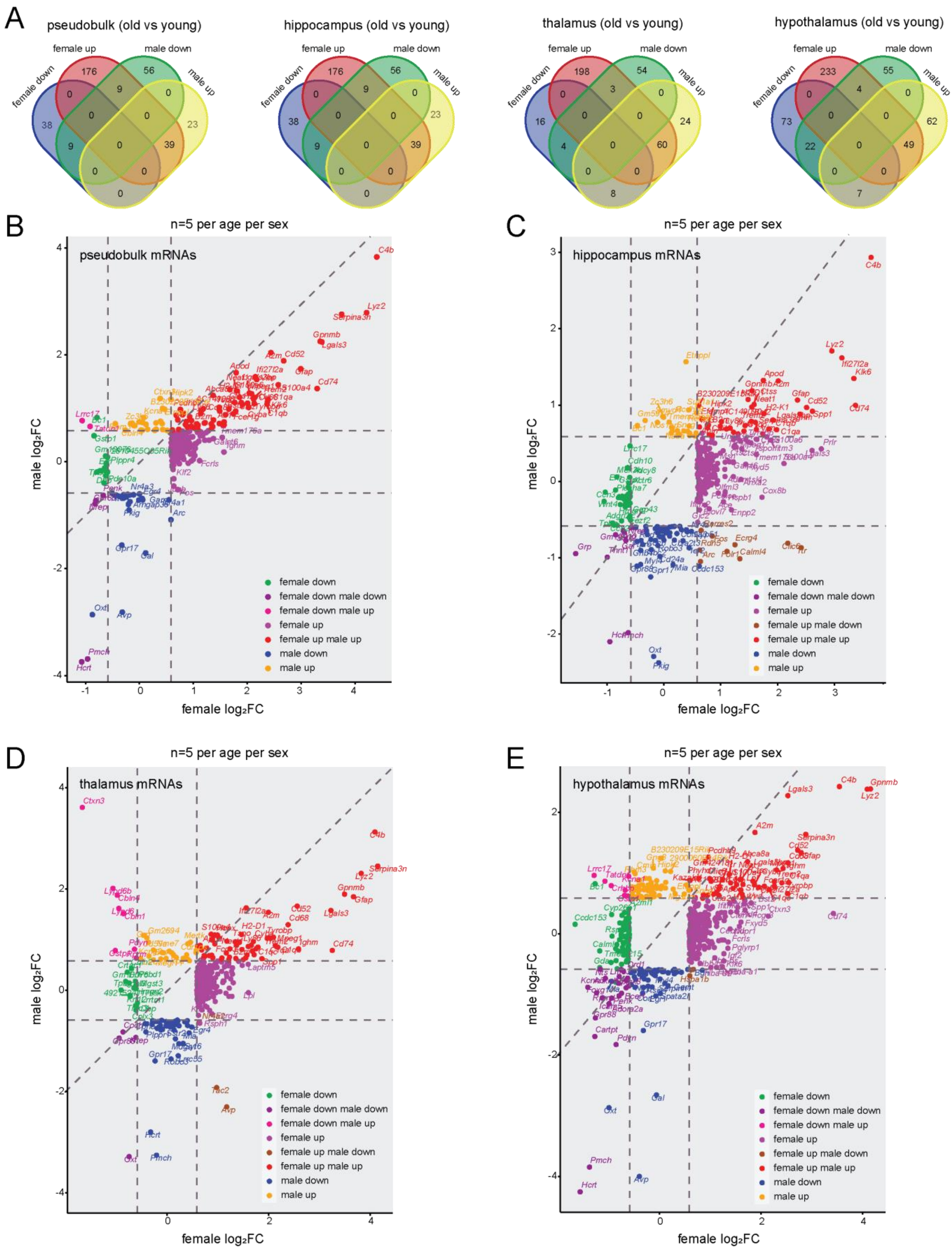

Supp. Figure 7: Age-related expression profiles differ based on sex across brain regions (related to Figs. 2-6).

(A) Venn diagrams showing overlap of 4 indicated groups of DARs in old vs. young, male and female mice in pseudobulk, hippocampus, thalamus and hypothalamus region. **(B)** Scatterplot showing significant DARs in old vs. young male and female mice (FDR <0.05, FC ≥1.5 or ≤ -1.5) in pseudobulk data. **(C)** Same as (B) except in hippocampus. **(D)** Same as (B) except in thalamus. **(E)** Same as (B) except in hypothalamus. For (A-E), some top up- or downregulated mRNAs are labeled. Source data are provided as a Source Data file.
