# Supplementary material for: Spatial transcriptomics of the aging mouse brain reveals origins of inflammation in the white matter
Source: Nat Commun. 2025 Apr 4;16:3231. doi: 10.1038/s41467-025-58466-2 (PMC11971433; doi:10.1038/s41467-025-58466-2)
Supplement: Supplementary file 2 — Description of Additional Supplementary Information [file 41467_2025_58466_MOESM2_ESM.pdf]

Supplementary Data Titles

File name:

Supp. Data 1: animal and antibody information

Description:

Sheet 1: spatial animal information

Sheet 2: index

Sheet 3: flow cytometry

Sheet 4: IF

Sheet 5: MRI

Sheet 6: antibodies for flow cytometry

Sheet 7: antibodies for IF

Sheet 8: antibodies for CUT&RUN

File name:

Supp. Data 2: sample slide information

Description:

Sheet 1: tissue

File name:

Supp. Data 3: 10109 filtered mRNA list

Description:

Sheet 1: 10109 filtered mRNA list

File name:

Supp. Data 4: DARs

Description:

Sheet 1: bulk (old vs young up)

Sheet 2: bulk (old vs young down)

Sheet 3: isocortex (old vs young up)

Sheet 4: isocortex (old vs young down)

Sheet 5: hypothalamus (old vs young up)

Sheet 6: hypothalamus (old vs young down)

Sheet 7: thalamus (old vs young up)

Sheet 8: thalamus (old vs young down)

Sheet 9: OLF.CTXsp (old vs young up)

Sheet 10: OLF.CTXsp (old vs young down)

Sheet 11: fiber tracts (old vs young up)

Sheet 12: fiber tracts (old vs young down)

Sheet 13: hippocampus (old vs young up)

Sheet 14: hippocampus (old vs young down)

Sheet 15: striatum/CNU (old vs young up)

Sheet 16: striatum/CNU (old vs young down)

File name:

Supp. Data 5: shared and unique DARs

Description:

Sheet 1: unique DARs

Sheet 2: shared DARs

Sheet 3: 39 shared (all 7 regions)

Sheet 3: 73 shared (5 regions)

Sheet 3: 183 shared (at least 2 regions)

Sheet 3: 91 shared (at least 2 regions)

File name:

Supp. Data 6: GSEA

Description:

Sheet 1: bulk (old vs young up)

Sheet 2: bulk (old vs young down)

Sheet 3: bulk (old vs middle up)

Sheet 4: bulk (old vs middle down)

Sheet 5: bulk (middle vs young up)

Sheet 6: bulk (middle vs young down)

Sheet 7: isocortex (old vs young up)

Sheet 8: isocortex (old vs young down)

Sheet 9: hypothalamus (old vs young up)

Sheet 10: hypothalamus (old vs young down)

Sheet 11: thalamus (old vs young up)

Sheet 12: thalamus (old vs young down)

Sheet 13: OLF.CTXsp (old vs young up)

Sheet 14: OLF.CTXsp (old vs young down)

Sheet 15: fiber tracts (old vs young up)

Sheet 16: fiber tracts (old vs young down)

Sheet 17: hippocampus (old vs young up)

Sheet 18: hippocampus (old vs young down)

Sheet 19: striatum/CNU (old vs young up)

Sheet 20: striatum/CNU (old vs young down)

File name:

Supp. Data 7: set1-4, WAM, DAM1, DAM2, SenNet mRNAs

Description:

Sheet 1: set1-4 (Safaiyan et al)

Sheet 2: WAM (Safaiyan et al)

Sheet 3: DAM1 (Keren-Shaul et al)

Sheet 4: DAM2 (Keren-Shaul et al)

Sheet 5: SenNet (Suryadevara et al)

File name:

Supp Data 8: RCTD

Description:

Sheet 1: all 25 cell types

File name:

Supp Data 9: male female DARs

Description:

Sheet 1: bulk

Sheet 2: fiber tracts

Sheet 3: hippocampus

Sheet 4: thalamus

Sheet 5: hypothalamus

File name:

Supp. Data 10: machine learning input

Description:

Sheet 1: cohort 1, features_chipseq_DE

Sheet 2: cohort 2, features_chipseq_DE
