# Supplementary material for: Spatial transcriptomics of the aging mouse brain reveals origins of inflammation in the white matter
Source: Nat Commun. 2025 Apr 4;16:3231. doi: 10.1038/s41467-025-58466-2 (PMC11971433; doi:10.1038/s41467-025-58466-2)
Supplement: Supplementary file 13 — Reporting Summary [file 41467_2025_58466_MOESM13_ESM.pdf]

nature portfolio

Corresponding author(s): Payel Sen

Last updated by author(s): Jan 30, 2025

Reporting Summary

Nature Portfolio wishes to improve the reproducibility of the work that we publish. This form provides structure for consistency and transparency in reporting. For further information on Nature Portfolio policies, see our Editorial Policies and the Editorial Policy Checklist.

Statistics

For all statistical analyses, confirm that the following items are present in the figure legend, table legend, main text, or Methods section.

n/a	Confirmed	
☐	☒	The exact sample size (*n*) for each experimental group/condition, given as a discrete number and unit of measurement
☐	☒	A statement on whether measurements were taken from distinct samples or whether the same sample was measured repeatedly
☐	☒	The statistical test(s) used AND whether they are one- or two-sided<br>*Only common tests should be described solely by name; describe more complex techniques in the Methods section.*
☐	☒	A description of all covariates tested
☐	☒	A description of any assumptions or corrections, such as tests of normality and adjustment for multiple comparisons
☐	☒	A full description of the statistical parameters including central tendency (e.g. means) or other basic estimates (e.g. regression coefficient) AND variation (e.g. standard deviation) or associated estimates of uncertainty (e.g. confidence intervals)
☐	☒	For null hypothesis testing, the test statistic (e.g. *F*, *t*, *r*) with confidence intervals, effect sizes, degrees of freedom and *P* value noted<br>*Give P values as exact values whenever suitable.*
☒	☐	For Bayesian analysis, information on the choice of priors and Markov chain Monte Carlo settings
☒	☐	For hierarchical and complex designs, identification of the appropriate level for tests and full reporting of outcomes
☐	☒	Estimates of effect sizes (e.g. Cohen's *d*, Pearson's *r*), indicating how they were calculated

Our web collection on statistics for biologists contains articles on many of the points above.

Software and code

Policy information about availability of computer code

Data collection	Spatial transcriptomics data were acquired using the Visium Spatial Transcriptomics kit (10X Genomics) followed by sequencing on the NovaSeq 6000 platform (Illumina). Data were processed using Space Ranger software (v2.0.1, v2.1.1, 10X Genomics) and Partek Flow (v10.0.23.0531, v11.0.23.1204, Partek Inc.). Immunofluorescence images were acquired using the all-in-one fluorescence microscope (Keyence, BZ-X710) or Zeiss LSM 710 confocal microscope followed by visualization using either Keyence BZ analyzer (v1.3.1.1), the Zeiss Zen image software (v3.1) or IMARIS (v9.9.0). MRI data were acquired using a 7T Bruker vertical bore MRI scanner equipped with a microimaging probe and a 10 mm RF coil and processed using using the TORTOISE V4 pipeline (https://github.com/eurotomania/TORTOISEV4). Flow cytometry data were acquired on a Cytoflex-LX Flow Cytometer (Beckman Coulter) and processed using CytExpert and FlowJo software (v10.8.1). CUT&RUN data were acquired using the CUT&RUN Assay Kit (cell signaling technology, 86652) followed by sequencing on the NextSeq 2000 platform using P2 and P4 XLEAP-SBS Reagent Kit (100-cycle) (Illumina). Data were demultiplexed using bcl-convert software (v4.1.5) , and then processed by Trim Galore (v0.6.7) to remove adapters. FASTQ quality was assessed using FASTQC (v0.11.9) and MultiQC(v1.14).
Data analysis	Spatial transcriptomics data were analyzed in Partek Flow (v10.0.23.0531, v11.0.23.1204 Partek Inc.).<br>Processed spatial transcriptomics data were analyzed in R Studio (v4.3.0, v4.4.0) using the package ggplot2, pheatmap, RCTD (v2.2.1), SPATA2 (v0.1.0).<br>Processed spatial transcriptomics data were analyzed in Python (v3.8) using the package SpatialDE (v1.1.0).<br>Processed spatial transcriptomics data were analyzed in GSEA software (v4.3.2).<br>Immunofluorescence images were analyzed in Image J software (Fiji).<br>MRI data were analyzed using the TORTOISE V4 pipeline (https://github.com/eurotomania/TORTOISEV4).<br>FACS data were analyzed in FlowJo software (v10.8.1).<br>CUT&RUN data were analyzed using Bowtie2 (v2.5.3), samtools (v1.19), bedtools (v2.30.0) and deepTools (v3.5.4)

Statistical analyses were analyzed in GraphPad Prism software (v9.4.1 and 10.2.1).
The code and pipeline for the analyses performed in this study are available at https://github.com/PSenlab/Wang_2025 and published in https://doi.org/10.5281/zenodo.14750569.

For manuscripts utilizing custom algorithms or software that are central to the research but not yet described in published literature, software must be made available to editors and reviewers. We strongly encourage code deposition in a community repository (e.g. GitHub). See the Nature Portfolio guidelines for submitting code & software for further information.

Data

Policy information about availability of data

All manuscripts must include a data availability statement. This statement should provide the following information, where applicable:

- Accession codes, unique identifiers, or web links for publicly available datasets
- A description of any restrictions on data availability
- For clinical datasets or third party data, please ensure that the statement adheres to our policy

Raw and processed spatial transcriptomics data and CUT&RUN data generated in this study are available through the NCBI Gene Expression Omnibus (GEO) repository under accession number GSE284202 (https://www.ncbi.nlm.nih.gov/geo/query/acc.cgi?acc=GSE284202). Source data are provided with this paper and also available at MendeleyData (https://doi.org/10.17632/96wzkvjm6n.1).

Research involving human participants, their data, or biological material

Policy information about studies with human participants or human data. See also policy information about sex, gender (identity/presentation), and sexual orientation and race, ethnicity and racism.

Reporting on sex and gender	N/A
Reporting on race, ethnicity, or other socially relevant groupings	N/A
Population characteristics	N/A
Recruitment	N/A
Ethics oversight	N/A

Note that full information on the approval of the study protocol must also be provided in the manuscript.

Field-specific reporting

Please select the one below that is the best fit for your research. If you are not sure, read the appropriate sections before making your selection.

☒ Life sciences ☐ Behavioural & social sciences ☐ Ecological, evolutionary & environmental sciences

For a reference copy of the document with all sections, see nature.com/documents/nr-reporting-summary-flat.pdf

Life sciences study design

All studies must disclose on these points even when the disclosure is negative.

Sample size	Samples sizes for each experiment are clearly written in the manuscript in the text and figure legends. No statistical methods were used to predetermine sample sizes; our sample sizes were determined based on commonly used sample sizes in published single-cell studies of the brain (e.g., Ximerakis et al, Nature Neuroscience 2019, PMID: 31551601; Hajdarovic et al, Nature Aging 2022, PMID: 36285248; Hahn et al, Cell 2023, PMID: 37591239; Allen et al, Cell 2023, PMID: 36580914).
Data exclusions	All exclusion criteria are established during data analysis and are delineated in the relevant sections of the Methods. In brief, for cohort 1 spatial transcriptomics experiments, the barcoded spots were filtered by counts at low.cutoff=1 and high.cutoff=38,855, detected genes at low.cutoff=1 and high.cutoff=8012, % mitochondrial counts at low.cutoff=0, high.cutoff=35%, % ribosomal counts at low.cutoff=0, high.cutoff=14%. Filtered genes with value ≤ 1.0 in at least 99 % of the cells were excluded. For cohort 2 spatial transcriptomics RNA-seq experiments, the barcoded spots were filtered by counts at low.cutoff=1 and high.cutoff=31,732, detected genes at low.cutoff=1 and high.cutoff=7515, % mitochondrial counts at low.cutoff=0, high.cutoff=48%, % ribosomal counts at low.cutoff=0, high.cutoff=18%. Filtered genes with value ≤ 1.0 in at least 99 % of the cells were excluded. For CUT&RUN experiments, due to insufficient cell numbers for transposition, library construction failed for one young female CEBPB, one old female CEBPB and two young female YY1 samples which were excluded from pooling and sequencing. For immunofluorescence, flow cytometry and MRI experiments, no data were excluded.
Replication	Main findings that are highlighted in the manuscript were successfully reproduced in a separate cohort and using independent methodologies (immunofluorescence, flow cytometry, and MRI).

Randomization	No randomization was used as there was no treatment group in our study. However, young, middle, and old mice were randomly assigned to the different sets of experiments (spatial transcriptomics, immunofluorescence, flow cytometry, CUT&RUN and MRI).
Blinding	Blinding was not possible, as knowledge of the experimental conditions was required during the data collection and analyses.

Reporting for specific materials, systems and methods

We require information from authors about some types of materials, experimental systems and methods used in many studies. Here, indicate whether each material, system or method listed is relevant to your study. If you are not sure if a list item applies to your research, read the appropriate section before selecting a response.

Materials & experimental systems

n/a	Involved in the study	
☐	☒	Antibodies
☒	☐	Eukaryotic cell lines
☒	☐	Palaeontology and archaeology
☐	☒	Animals and other organisms
☒	☐	Clinical data
☒	☐	Dual use research of concern
☒	☐	Plants

Methods

n/a	Involved in the study	
☐	☒	ChIP-seq
☐	☒	Flow cytometry
☐	☒	MRI-based neuroimaging

Antibodies

Antibodies used

All antibodies used in this study are listed in Supplementary Data 1.

For flow cytometry:
BUV395 Rat Anti-Mouse CD45 antibody (BD Biosciences #564279) (0.2ug in 50ul volume)
https://www.bdbiosciences.com/en-us/products/reagents/flow-cytometry-reagents/research-reagents/single-color-antibodies-ruo/buv395-rat-anti-mouse-cd45.564279
Pacific Blue™ anti-mouse Ly-6G/Ly-6C (Gr-1) antibody (Biolegend #108430) (0.5ug in 50ul volume)
https://www.biolegend.com/en-ie/products/pacific-blue-anti-mouse-ly-6g-ly-6c-gr-1-antibody-4143?GroupID=BLG4876
Brilliant Violet 510™ anti-mouse/human CD11b antibody (Biolegend #101263) (0.2ug in 50ul volume)
https://www.biolegend.com/en-us/products/brilliant-violet-510-anti-mouse-human-cd11b-antibody-7993?GroupID=BLG10552
Brilliant Violet 650™ anti-mouse Ly-6G antibody (Biolegend #127641) (0.2ug in 50ul volume)
https://www.biolegend.com/en-us/products/brilliant-violet-650-anti-mouse-ly-6g-antibody-11981
APC anti-mouse CD115 (CSF-1R) antibody (Biolegend #135510) (0.2ug in 50ul volume)
https://www.biolegend.com/en-us/products/apc-anti-mouse-cd115-csf-1r-antibody-6336?GroupID=BLG8949
PE/Dazzle™ 594 anti-mouse CD183 (CXCR3) antibody (Biolegend #126533) (0.2ug in 50ul volume)
https://www.biolegend.com/fr-ch/products/pe-dazzle-594-anti-mouse-cd183-cxcr3-antibody-12482
MHC Class II I-Ab Monoclonal Antibody (AF6-120.1), FITC, eBioscience™ (Invitrogen #11-5320-82) (0.5ug in 50ul volume)
https://www.thermofisher.com/antibody/product/MHC-Class-II-I-Ab-Antibody-clone-AF6-120-1-Monoclonal/11-5320-82
BUV661 Rat Anti-Mouse F4/80 antibody (BD Biosciences #750643) (0.2ug in 50ul volume)
https://www.bdbiosciences.com/en-es/products/reagents/flow-cytometry-reagents/research-reagents/single-color-antibodies-ruo/buv661-rat-anti-mouse-f4-80.750643
Brilliant Violet 605™ anti-mouse Ly-6C antibody (Biolegend #128306) (0.05ug in 50ul volume)
https://www.biolegend.com/en-us/products/brilliant-violet-605-anti-mouse-ly-6c-antibody-8727?GroupID=BLG7242

For immunofluorescence:
Iba1/AIF-1(E4O4W) XP rabbit mab (Cell Signaling Technology #17198S) (1:100 dilution)
https://www.cellsignal.com/products/primary-antibodies/iba1-aif-1-e4o4w-xp-rabbit-mab/17198
Anti-GFAP antibody (Abcam #ab53554) (1:1000 dilution)
https://www.abcam.com/products/primary-antibodies/gfap-antibody-ab53554.html
Recombinant Anti-Myelin Basic Protein antibody (Abcam #ab7349) (1:5000 dilution)
https://www.abcam.com/products/primary-antibodies/myelin-basic-protein-antibody-12-ab7349.html
Purified anti-mouse/human Mac-2 (Galectin-3) Antibody (Biolegend #125402) (1:1000 dilution)
https://www.biolegend.com/en-us/products/purified-anti-mouse-human-mac-2-galectin-3-antibody-4935?GroupID=GROUP20
CNPase Antibody (G-6) (Santa cruz #sc-166063) (1:1000 dilution)
https://www.scbt.com/p/cnpase-antibody-g-6
Goat anti-Rat IgG (H+L) Cross-Adsorbed Secondary Antibody, Alexa Fluor™ 488 (Thermo Fisher #A11006) (1:1000 dilution)
https://www.thermofisher.com/antibody/product/Goat-anti-Rat-IgG-H-L-Cross-Adsorbed-Secondary-Antibody-Polyclonal/A-11006
Rabbit anti-Goat IgG (H+L) Cross-Adsorbed Secondary Antibody, Alexa Fluor™ 488 (Thermo Fisher #A11078) (1:1000 dilution)
https://www.thermofisher.com/antibody/product/Rabbit-anti-Goat-IgG-H-L-Cross-Adsorbed-Secondary-Antibody-Polyclonal/A-11078
Goat anti-Rabbit IgG (H+L) Cross-Adsorbed Secondary Antibody, Alexa Fluor™ 568 (Thermo Fisher #A11011) (1:1000 dilution)
https://www.thermofisher.com/antibody/product/Goat-anti-Rabbit-IgG-H-L-Cross-Adsorbed-Secondary-Antibody-Polyclonal/A-11011
Donkey anti-Mouse IgG (H+L) Highly Cross-Adsorbed Secondary Antibody, Alexa Fluor™ 568 (Thermo Fisher #A10037) (1:1000 dilution)

https://www.thermofisher.com/antibody/product/Donkey-anti-Mouse-IgG-H-L-Highly-Cross-Adsorbed-Secondary-Antibody-Polyclonal/A10037

For CUT&RUN:
C/EBP beta Antibody (H-7) (Santa Cruz # sc-7962) (1.5ug in 50ul volume)
https://www.scbt.com/p/c-ebp-beta-antibody-h-7?srsltid=AfmBOoqWzhYBBlCY3tFaljvqLZ1ISMiyhxBg15w27rIkfoOXwVkIMNUn
YY1 (D5D9Z) Rabbit mAb (Cell Signaling Technology #46395) (1.5ug in 50ul volume)
https://www.cellsignal.com/products/primary-antibodies/yy1-d5d9z-rabbit-mab/46395?srsltid=AfmBOopkX-70hArUa8MWo2EsE4B4V7Ku8efiqVR7m2JEuHJcEuZzUAUf
Rabbit IgG, polyclonal - Isotype Control (ChIP Grade) (Abcam #ab171870) (1.5ug in 50ul volume)
https://www.abcam.com/en-us/products/primary-antibodies/rabbit-igg-polyclonal-isotype-control-chip-grade-ab171870

Validation

All the antibodies used are well characterized commercial antibodies. For each antibody, the specificity has been tested by the manufacturer and verified independently by previous published studies. Validation profiles and relevant citations can be found in the links provided.

Animals and other research organisms

Policy information about studies involving animals; ARRIVE guidelines recommended for reporting animal research, and Sex and Gender in Research

Laboratory animals

Details of all animals used in this study are described in Supplementary Data 1.

For spatial transcriptomics, male and female C57BL6/JN mice (https://ros.nia.nih.gov/) were used. Young mice were 11 weeks old, middle-aged mice were 57-58 weeks old and old mice were 125-127 weeks old.

For flow cytometry, male and female C57BL6/JN mice (https://ros.nia.nih.gov/) were used. Young mice were 14-16 weeks old and old mice were 102-110 weeks old.

For immunofluorescence, only female C57BL6/JN mice (https://ros.nia.nih.gov/) were used. Young mice were 12-16 weeks old and old mice were 85-86 weeks old.

For MRI, only female C57BL6/JN mice (https://ros.nia.nih.gov/) were used. Young mice were 12 weeks old and old mice were 103 weeks old.

For CUT&RUN, female and male C57BL6/JN mice (https://ros.nia.nih.gov/) were used. Young mice were at 14 (female) and 16 (male) weeks of age, and old mice were 88 (female) and 90 (male) weeks of age.

Wild animals

This study did not involve wild animals.

Reporting on sex

Sex was considered in spatial transcriptomics and flow cytometry experiments. Since female mice had a stronger pro-inflammatory signature in our study, validatory immunofluorescence and MRI experiments were performed only in female mice. Animal sex information for each experiment is provided in Supplementary Data 1.

Field-collected samples

The study did not involve field-collected samples.

Ethics oversight

This study was approved by the Animal Care and Use Committee of the NIA in Baltimore, MD under Animal Study Protocol number 481-LGG-2025. Routine tests are performed to ensure that mice are pathogen-free and sentinel cages are maintained and tested according to American Association for Accreditation of Laboratory Animal Care (AAALAC) criteria.

Note that full information on the approval of the study protocol must also be provided in the manuscript.

Plants

Seed stocks

N/A

Novel plant genotypes

N/A

Authentication

N/A

ChIP-seq

Data deposition

☒ Confirm that both raw and final processed data have been deposited in a public database such as GEO.

☐ Confirm that you have deposited or provided access to graph files (e.g. BED files) for the called peaks.

Data access links<br>*May remain private before publication.*	Raw and processed spatial transcriptomics data and CUT&RUN data generated in this study are available through the NCBI Gene Expression Omnibus (GEO) repository under accession number GSE284202.
Files in database submission	For spatial transcriptomics data (n=24 in cohort 1 and n=20 in cohort 2), raw fastq files and processed filtered_feature_bc_matrix.h5 and image files in tif format are provided. For CUT&RUN, raw fastq files and processed bigwig files are provided.
Genome browser session<br>(e.g. UCSC)	Not provided due to restrictions in NIH server access. bigWig files are provided as GEO submission (see above).

Methodology

Replicates	For CUT&RUN, we used microglia derived from either young or old mouse brains. Microglia from 2 mice were pooled into one biological replicate. 3 young and 3 old biological replicates were included in CUT&RUN.
Sequencing depth	The library was sequenced twice using P2 and P4 100 cycle kits . Total paired end reads were 901.26M and 3640.36M from P2 and P4 respectively. In total, we got a sequencing depth of 107.16M paired end reads per sample.
Antibodies	C/EBP beta Antibody (H-7)(Santa Cruz # sc-7962)<br>https://www.scbt.com/p/c-ebp-beta-antibody-h-7?srsltid=AfmBOoqWzhYBBlCY3tFaljvqLZ1ISMiyhxBg15w27rIkfoOXwVkIMNUn<br>YY1 (D5D9Z) Rabbit mAb(Cell Signaling Technology #46395)<br>https://www.cellsignal.com/products/primary-antibodies/yy1-d5d9z-rabbit-mab/46395?<br>srsltid=AfmBOopkX-70hArUa8MWo2EsE4B4V7Ku8efiqVR7m2JEuHJcEuZzUAUf<br>Rabbit IgG, polyclonal - Isotype Control (ChIP Grade) (Abcam #ab171870)<br>https://www.abcam.com/en-us/products/primary-antibodies/rabbit-igg-polyclonal-isotype-control-chip-grade-ab171870
Peak calling parameters	N/A
Data quality	N/A
Software	Sequencing reads were demultiplexed generating compressed FASTQ files by bcl-convert (v4.1.50).<br>Paired-end reads were trimmed using Trim Galore (v0.6.7) to remove adapters and qualities of the FASTQs assessed using FASTQC (v0.11.9) and MultiQC (v1.14).<br>SAM output files from Bowtie2 were filtered to retain alignments with a minimum mapping quality of 2 using samtools (v1.19) and aligned reads mapping to ENCODE blacklist regions were removed using intersect function in bedtools (v2.30.0).<br>RPKM normalized bigWig files were generated using the bamCoverage function in deepTools (v3.5.4).

Flow Cytometry

Plots

Confirm that:

☒ The axis labels state the marker and fluorochrome used (e.g. CD4-FITC).

☒ The axis scales are clearly visible. Include numbers along axes only for bottom left plot of group (a 'group' is an analysis of identical markers).

☒ All plots are contour plots with outliers or pseudocolor plots.

☒ A numerical value for number of cells or percentage (with statistics) is provided.

Methodology

Sample preparation	For the flow cytometry experiments, single cell suspensions derived from either young or old mouse brains were prepared using the Adult Brain Dissociation kit (Miltenyi Biotec,130-107-677).
Instrument	Cytoflex-LX Flow Cytometer (Beckman Coulter).
Software	Data were analyzed using the CytExpert and FlowJo software (v10.8.1).
Cell population abundance	N/A

Gating strategy

Neutrophils were first gated on forward scatter (FSC-A) and side scatter (SSC-A), followed by gating on single cells, live cells and indicated markers in Fig. S6C.

☒ Tick this box to confirm that a figure exemplifying the gating strategy is provided in the Supplementary Information.

Magnetic resonance imaging

Experimental design

Design type	Ex vivo MRI
Design specifications	N/A
Behavioral performance measures	N/A

Acquisition

Imaging type(s)	diffusion
Field strength	7
Sequence & imaging parameters	For dMRI, 261 image volumes were acquired for each specimen using a 3D echo planar imaging pulse sequence with the following parameters: echo time =42 ms; repetition time=800 ms; number of segments=10; and isotropic voxel dimension=75 µm.
Area of acquisition	Whole brain

Diffusion MRI ☒ Used ☐ Not used

Parameters: Data were acquired using a multishell acquisition with six directions and three repetitions for b=200, 500, and 1000 s/mm^2, 32 directions and 1 repetition for b=1700 and 3800 s/mm^2, 56 directions and 1 repetition for b=6700 s/mm^2, and 87 directions and 1 repetition for b=10,000 s/mm^2. An additional b=0 s/mm2 image was also acquired with reversed phase-encoding for distortion correction purposes. All dMRI data were acquired using δ=3 ms and Δ=20 ms.

Preprocessing

Preprocessing software	Diffusion MRI preprocessing was performed using the TORTOISE V4 pipeline. The data was first denoised using the MP-PCA denoising technique and subsequently corrected for eddy-currents distortions. During this process, each diffusion weighted image was initially quadratically registered to an average b=0 s/mm^2 to image, and subsequently registered to a synthetic image generated using the same bval/bvec with either the DTI model (for b-values up to 6000 s/mm^2) or the MAP-MRI model (for b-values larger than 6000 s/mm^2). Center frequency drifts were then estimated using a linear regression model and applied to all data. The final step in processing was susceptibility distortion correction with the DRBUDDI technique, which used both the blip-up and blip-down dMRI data along with the corresponding T2W image for correct. The final dMRI data was generated by concatenating the blip-up and -down datasets.
Normalization	The Turone atlas [Barriere, D. A. Turone Mouse Brain Template and Atlas. NeuroImaging Tools and Research Collaboratory https://www.nitrc.org/projects/tmbta_2019/] was used as a common template for registration. Nonlinear registration was performed to transform each brain into template common space using the greedy diffeomorphic algorithm (greedy, https://github.com/pyushkevich/greedy).
Normalization template	The Turone atlas
Noise and artifact removal	See "Preprocessing software" above
Volume censoring	See "Preprocessing software" above

Statistical modeling & inference

Model type and settings	Two-sample t-test
Effect(s) tested	Quantitative comparisons of dMRI parameters averaged across the whole-brain WM between the young and old groups.

Specify type of analysis: ☐ Whole brain ☒ ROI-based ☐ Both

Anatomical location(s): The Turone Mouse Brain Template and Atlas was used: probabilistic WM atlas thresholded at 0.9.

Statistic type for inference (See Eklund et al. 2016)	*Specify voxel-wise or cluster-wise and report all relevant parameters for cluster-wise methods.*

nature portfolio | reporting summary

Correction	t-test with Welch's correction

Models & analysis

n/a	Involved in the study
☒	☐ Functional and/or effective connectivity
☒	☐ Graph analysis
☒	☐ Multivariate modeling or predictive analysis
